# CIRBP-OGFR axis safeguards against cardiomyocyte apoptosis and cardiotoxicity induced by chemotherapy

**DOI:** 10.7150/ijbs.69655

**Published:** 2022-04-11

**Authors:** Cihang Liu, Xiaolei Cheng, Junyue Xing, Jun Li, Zhen Li, Dongdong Jian, Ying Wang, Shixing Wang, Ran Li, Wanjun Zhang, Dongxing Shao, Xiaohan Ma, Xiru Chen, Jia Shen, Chao Shi, Zhiping Guo, Wengong Wang, Taibing Fan, Lin Liu, Hao Tang

**Affiliations:** 1 Department of Biochemistry and Molecular Biology, Beijing Key Laboratory of Protein Posttranslational Modifications and Cell Function, School of Basic Medical Sciences, Peking University Health Science Center, 38 Xueyuan Road, Beijing 100191, China.; 2 National Health Commission Key Laboratory of Cardiovascular Regenerative Medicine, Heart Center of Henan Provincial People's Hospital, Central China Fuwai Hospital of Zhengzhou University, Fuwai Central China Cardiovascular Hospital & Central China Branch of National Center for Cardiovascular Diseases, Zhengzhou, Henan, 451464, China.; 3 Department of Children's Heart Center, Henan Provincial People's Hospital, Department of Children's Heart Center of Central China Fuwai Hospital, Henan Key Medical Laboratory of Tertiary Prevention and Treatment for Congenital Heart Disease, Central China Fuwai Hospital of Zhengzhou University, Zhengzhou, Henan, 451464, China.; 4 Henan Key Laboratory of Chronic Disease Management, Department of Health Management Center, Henan Provincial People's Hospital, Department of Health Management Center of Central China Fuwai Hospital, Central China Fuwai Hospital of Zhengzhou University, Zhengzhou, Henan, 451464, China.; 5 Department of Cardiology, Henan Provincial People's Hospital, Department of Cardiology of Central China Fuwai Hospital, Henan Key Laboratory for Coronary Heart Disease Prevention and Control, Central China Fuwai Hospital of Zhengzhou University, Zhengzhou, Henan, 451464, China.; 6 State Key Laboratory of Cardiovascular Disease, Fuwai Hospital, National Center for Cardiovascular Diseases, Chinese Academy of Medical Sciences and Peking Union Medical College, Beijing 100037, China.; 7 Department of Hematology, Henan Provincial People's Hospital, Zhengzhou, Henan, 450003, China.; 8 Henan Key Laboratory of Molecular Pathology, Department of Molecular Pathology, The Affiliated Cancer Hospital of Zhengzhou University, Zhengzhou, Henan, 450008, China.

**Keywords:** CIRBP, Cardioprotection, OGFR, Chemotherapy-induced cardiotoxicity, Cardiomyocyte apoptosis

## Abstract

Cold-inducible RNA-binding protein (CIRBP) is documented to be required for maintaining cardiac function, however, its role in chemotherapy-induced cardiotoxicity remains obscured. Herein, we report that CIRBP decreases cardiomyocyte apoptosis and attenuates cardiotoxicity through disrupting OGF-OGFR signal. CIRBP deficiency is involved in diverse chemotherapeutic agents induced cardiomyocyte apoptosis. Delivery of exogenous CIRBP to the mouse myocardium significantly mitigated doxorubicin-induced cardiac apoptosis and dysfunction. Specifically, OGFR was identified as a downstream core effector responsible for chemotherapy-induced cardiomyocyte apoptosis. CIRBP was shown to interact with OGFR mRNA and to repress OGFR expression by reducing mRNA stability. CIRBP-mediated cytoprotection against doxorubicin-induced cardiac apoptosis was demonstrated to largely involve OGFR repression by CIRBP. NTX as a potent antagonist of OGFR successfully rescued CIRBP ablation-rendered susceptibility to cardiac dyshomeostasis upon exposure to doxorubicin, whereas another antagonist ALV acting only on opioid receptors did not. Taken together, our results demonstrate that CIRBP confers myocardium resistance to chemotherapy-induced cardiac apoptosis and dysfunction by dampening OGF/OGFR axis, shedding new light on the mechanisms of chemo-induced cardiotoxicity and providing insights into the development of an efficacious cardioprotective strategy for cancer patients.

## Introduction

Owing to the development of anti-neoplastic therapies, the life expectancy of cancer patients has been greatly extended in the past few decades. However, the living quality of these cancer survivors appears not to be improved due to various cardiovascular complications, which are commonly recognized as therapy-produced cardiotoxicity 1. Chemotherapy, a well-established treatment regimen for cancers, has been widely reported of bringing about cardiotoxicity, such as left ventricular dysfunction, dilated cardiomyopathy, myocardial ischemia, hypertension, and congestive heart failure (CHF) 2. Albeit with diverse pathogenesis of distinct chemotherapeutics in triggering cardiotoxicity, involving oxidative stress of reactive oxygen species (ROS), cumulative DNA damage, disturbance of intracellular calcium homeostasis and mitochondrial dysfunction, pro-apoptotic signaling activation seems to be a common downstream process 1, 2. Indeed, massive cardiomyocyte apoptosis has been observed in the myocardium in cancer patients with CHF treated with the anticancer drug doxorubicin (DOX) 3, 4. Moreover, previous studies have demonstrated that suppression of cardiomyocyte apoptosis conspicuously mitigates chemotherapeutic agent-induced cardiac dysfunction 5, 6, underscoring the role of apoptosis in cardiotoxicity. Hence, elucidating the shared pathway by which multiple chemotherapeutic drugs regulate cardiomyocyte apoptosis will unambiguously be beneficial for developing an efficacious cardioprotective strategy for patients receiving chemotherapy.

Cold-inducible RNA-binding protein (CIRBP) was discovered as a stress protein, increasing not only in response to mild hypothermia, but also to multifarious cellular stresses including ultraviolet light radiation, hypoxia, and osmotic and ischemic conditions 7. Mounting evidence suggests that CIRBP assists cells in adapting to damaging environmental changes by stabilizing specific mRNAs and facilitating their translation, thus enhancing cell survival and proliferation and inhibiting apoptosis and senescence 7. The cytoprotective function of CIRBP has been explored under a plethora of physiological and pathological conditions, and CIRBP was proposed more recently to mediate hypothermic cardioprotection on the cardioplegia-arrested heart in patients receiving surgery. Chronic hypoxia characterized in cyanotic congenital heart disease rendered CIRBP promoter hypermethylation in turn reducing CIRBP hypothermic elicitation and crippling its cytoprotective potency, indicative of the importance of CIRBP in cardioprotection 8. It has been suggested that CIRBP-mediated cytoprotection against apoptosis under cold shock stress was attributed in part to the activation of anti-apoptotic signaling, such as the MAPK/ERK_1/2_ cascade and NF-κB pathway 9-11. However, very little is known about the role CIRBP plays in cardiac apoptosis and cardiotoxicity evoked by chemical agents.

In this work, we found that CIRBP plays a crucial role in safeguarding myocardium against cardiac apoptosis and cardiotoxicity throughout chemotherapy. Opioid growth factor receptor (OGFR), an essential part constituting OGF/OGFR signal pathway for cellular survival, was identified as the downstream effector accounting for CIRBP loss-aggravated cardiac apoptosis. OGFR is a non-canonical, perinuclear opioid receptor that does not share structural homology with canonical δ, κ, and μ opioid receptors, and OGF (also known as methionine enkephalin, MENK) is a tonic inhibitory peptide ligand of OGFR 12, 13. Our results showed that CIRBP reduced the stability of OGFR mRNA by binding to it. CIRBP-mediated cardioprotection against apoptosis depends primarily on the interaction between CIRBP and OGFR mRNA. Unlike alvimopan (ALV), a pan-opioid receptor antagonist that simultaneously targets δ, κ, and μ opioid receptors 14, the potent OGFR blocker naltrexone (NTX), which has suppressive effects on some opioid receptors as well 15, 16, successfully rescued CIRBP ablation-rendered cardiac susceptibility to apoptosis, further stressing the role of OGFR suppression by CIRBP in protecting against chemotherapy-induced cardiotoxicity. Together, these findings indicate that OGFR augmentation resulting from CIRBP deficiency bolsters OGF/OGFR signaling, thereby predisposing the myocardium to cardiac apoptosis and toxicity during chemotherapy. Restoration of CIRBP and/or blunting OGF/OGFR signal are potential treatments for cardiotoxicity acquired from cancer therapies.

## Methods

Detailed methods are described in the Supplementary materials.

### Animal studies and ethics

All animal care and experimental procedures were approved by the Animal Care and Use Committee of Zhengzhou University and performed in compliance with European Communities Council Directive 86/609/EEC and 2010/63/EU for the protection of animals used for experimental purposes.

### Statistical analysis

Statistical analysis was performed using the SPSS 18.0 software (SPSS, Inc., Chicago, IL, USA). The data are expressed as the mean ± SD. Student's t test (two-tailed) was used to compare the differences between two groups. When comparing multiple groups, one-way ANOVA followed by Tukey-Kramer multiple comparisons was used. *, *p* < 0.05; **, *p* < 0.01; ***, *p* < 0.001; ns, no significant.

## Results

### CIRBP reduction is involved in cardiomyocyte apoptosis and cardiotoxicity induced by chemotherapeutics

**Figure 1:**
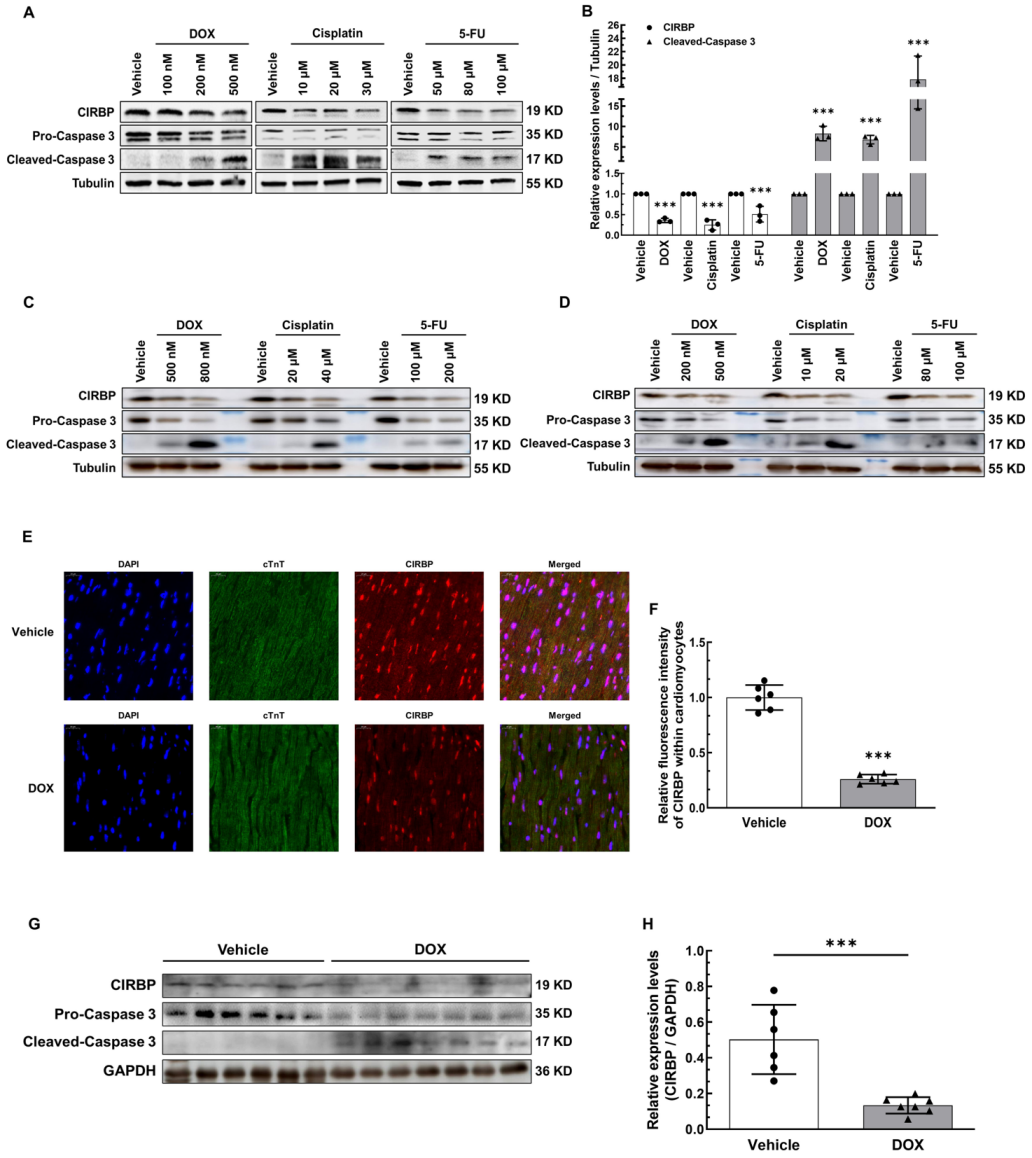
** Reduction in cold-inducible RNA-binding protein (CIRBP) expression is involved in cardiomyocyte apoptosis and cardiotoxicity induced by chemotherapeutics. A,** Representative immunoblots assessing apoptosis induced by three conventional chemotherapeutics (doxorubicin (DOX), cisplatin, and 5-fluorouracil (5-FU)) in immortalized human ventricular myocytes (AC16). **B,** Densitometric quantification of the relative protein levels of CIRBP and Cleaved-Caspase 3 in A after treatment with 500 nM DOX, 30 µM cisplatin, and 100 µM 5-FU. **C,** Representative immunoblots assessing apoptosis of human induced pluripotent stem cell-derived cardiomyocytes (hiPSC-CMs) induced by the aforementioned chemotherapeutics. **D,** Representative immunoblots assessing apoptosis of neonatal rat ventricular myocytes (NRVMs) induced by the aforementioned chemotherapeutics. E, Representative immunofluorescence images of heart tissue sections from mice receiving vehicle or DOX. Nuclei, DAPI (blue); cardiomyocytes, cardiac troponin T (cTnT) (green); CIRBP (red). Scale bar, 20 µm. **F,** Quantification of the relative CIRBP fluorescence intensity in 6 microscopic fields from 3 hearts per group in **E. G,** Immunoblot analysis of protein expression in heart tissues from mice receiving vehicle (n = 6) or DOX (n = 7). **H,** Densitometric quantification of CIRBP protein levels in G. The data are presented as the mean ± SD of from three independent experiments unless otherwise specified. And statistical significance was analysed using Student's *t*-test. ***, *p* < 0.001.

Given the fact that CIRBP can protect against cellular stress, we attempted to explore whether CIRBP is involved in chemotherapeutic drug-induced cardiomyocyte apoptosis. We initially treated human immortalized ventricular cardiomyocytes AC16 and T0519 (purchased from Abm Inc., Canada) with three typical chemotherapeutics (DOX, cisplatin, and 5-FU) to induce apoptosis. Not surprisingly, CIRBP expression decreased drastically during cell apoptosis elicited by all three agents (AC16, Figure 1A and B; T0519, Figure S1A). Meanwhile, both the beating human induced pluripotent stem cell-derived cardiomyocytes (hiPSC-CMs) and relatively mature neonatal rat ventricular myocytes (NRVMs) were utilized further to corroborate this alteration (hiPSC-CMs, Figure 1C; NRVMs, Figure 1D). Moreover, a preclinical mouse model that closely mimics clinical DOX-induced cardiotoxicity, as documented previously 17, 18, was generated to investigate cardiac CIRBP expression in response to DOX insult. Compared with control mice injected with normal saline (vehicle), mice treated with DOX exhibited impairment of cardiac function with dramatic decreases in EF and FS (Figure S1B), a reduction in the heart weight to tibia length (HW/TL) ratio (Figure S1C), increases in the serum levels of heart injury and heart failure markers (cTNT, LDH, CK-MB, and NT-proBNP) (Figure S1D), and massive cardiomyocyte apoptosis (Figure S1E and F). An obvious reduction in CIRBP expression in the myocardium (~ 27.13% of that in vehicle-treated mice, Figure 1E and F), the heart tissues (~ 26.54% of that in vehicle-treated mice, Figure 1G and H), and the apoptotic cardiac cells (Figure S1G) was observed in DOX-treated mice compared with vehicle-treated mice. These results indicate that chemotherapy-induced cardiac apoptosis and dysfunction likely involves CIRBP deficiency.

### CIRBP ablation exacerbates DOX-induced cardiomyocyte apoptosis

**Figure 2:**
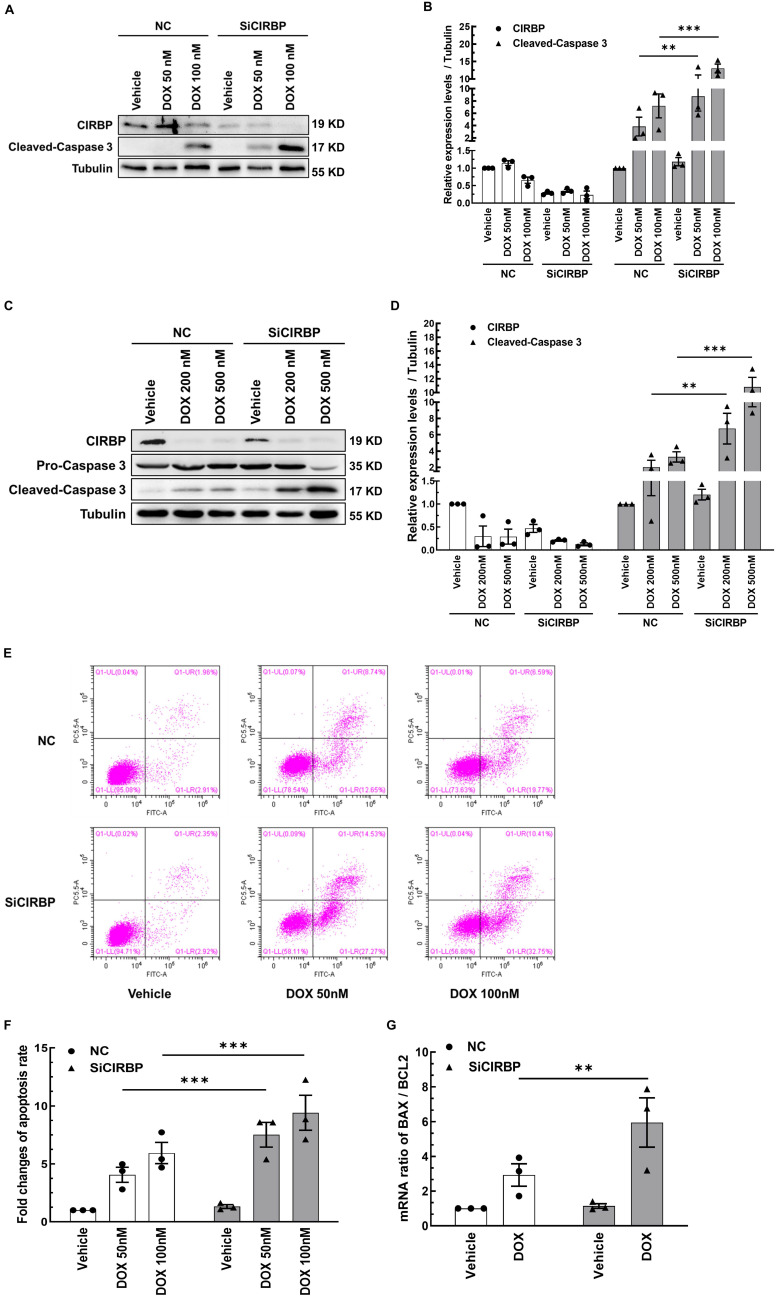
** CIRBP ablation exacerbates DOX-induced cardiomyocyte apoptosis. A,** Representative immunoblots assessing DOX-induced apoptosis of AC16 cells transfected with negative control siRNA (NC) or CIRBP siRNA (SiCIRBP). **B,** Densitometric quantification of the relative protein levels of CIRBP and Cleaved-Caspase 3 in A. Vehicle-treated cells transfected with NC siRNA served as controls for quantification. **C,** Representative immunoblots assessing DOX-induced apoptosis of NRVMs transfected with NC or SiCIRBP. **D,** Densitometric quantification of CIRBP and Cleaved-Caspase 3 protein levels in C. Vehicle-treated cells transfected with NC served as controls for quantification. **E,** Flow cytometry analysis of apoptosis of the cells described in A. **F,** Statistical analysis of the apoptosis rate in **E. G,** Quantification of the relative mRNA levels of BAX and BCL2 (BAX/BCL2) for assessment of DOX-induced apoptosis of AC16 cells transfected with NC or SiCIRBP. The data are presented as the mean ± SD of three independent experiments. And statistical significance was analysed by one-way ANOVA followed by Tukey-Kramer multiple comparisons **, *p* < 0.01; ***, *p* < 0.001.

To determine whether CIRBP participates in protecting cardiomyocytes from chemotherapy-induced apoptosis, CIRBP was knocked down in both AC16 cells and NRVMs; then, the cells were exposed to different doses of DOX (50 nM or 100 nM DOX for AC16 cells, 200 nM or 500 nM DOX for NRVMs) to ignite the apoptosis program. Our results showed that CIRBP loss markedly aggravated DOX-induced apoptosis, as indicated by an increase in the expression of cleaved caspase 3 compared with that in the group treated with the same dose of DOX (AC16, Figure 2A and B; NRVMs, Figure 2C and D), an increased percentage of apoptotic cells (Figure 2E and F), and a robust increase in the BAX/BCL2 ratio (Figure 2G). These data suggest that CIRBP robustly protects against cardiomyocyte apoptosis induced by DOX.

### CIRBP mitigates mouse cardiotoxicity in response to DOX insult

**Figure 3:**
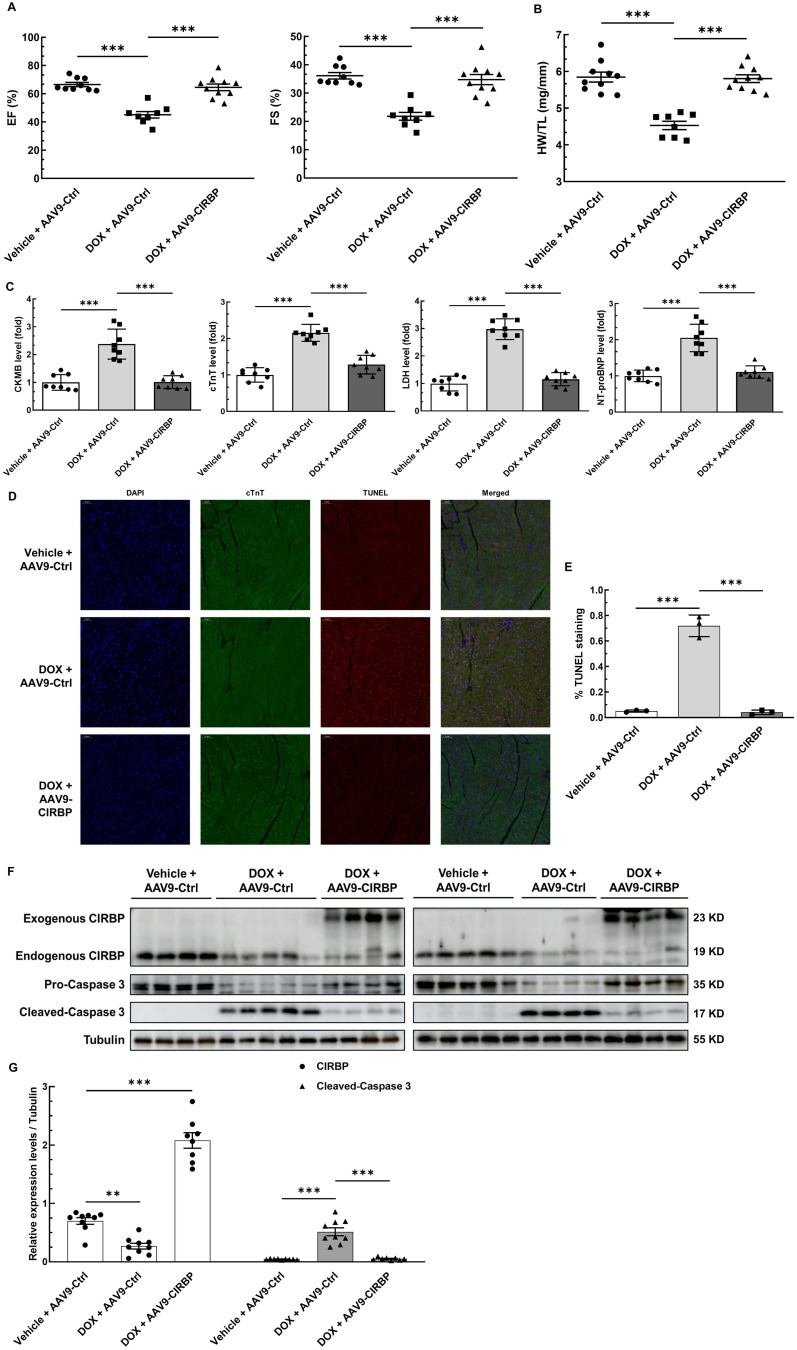
** CIRBP mitigates mouse myocardial apoptosis and cardiotoxicity to protect against DOX insult. A,** Echocardiographic analysis of cardiac function in mice pre-injected with an adeno-associated virus 9 (AAV9) vector harbouring a control (AAV9-Ctrl) or CIRBP (AAV9-CIRBP)-expressing cassette in the presence or absence of DOX (vehicle or DOX). EF, ejection fraction; FS, fractional shortening. Vehicle + AAV9-Ctrl, n = 9; DOX + AAV9-Ctrl, n = 8; DOX + AAV9-CIRBP, n = 10. **B,** Statistical analysis of the heart weight/tibia length (HW/TL) ratio of the mice in A. **C,** Biochemical determination of the relative serum levels of creatine kinase-myocardial band (CK-MB), cTnT, lactate dehydrogenase (LDH), and N-terminal pro-B type natriuretic peptide (NT-proBNP) to assess cardiac injury in the mice in A. **D,** Representative TUNEL staining images of heart tissue sections from the mice in A. Nuclei, DAPI (blue); cardiomyocytes, cTnT (green); TUNEL (red). Scale bar, 50 µm. **E,** Quantification of TUNEL staining in 3 microscopic fields from the hearts of the mice in A. **F,** Immunoblot analysis of protein levels in heart tissues from the mice in A. **G,** Densitometric quantification of CIRBP and Cleaved-Caspase 3 expression levels in F. Vehicle + AAV9-Ctrl, n = 9; DOX + AAV9-Ctrl, n = 9; DOX + AAV9-CIRBP, n = 8. The data are presented as the mean ± SD. And statistical significance was analysed by one-way ANOVA followed by Tukey-Kramer multiple comparisons. ***, *p* < 0.001.

To investigate the role of CIRBP in protecting the heart from chemotherapy-induced cardiac apoptosis and toxicity, we specifically overexpressed CIRBP in the mouse myocardium via injection of an AAV9 vector carrying a CIRBP-expressing cassette under the control of the cTnT promoter. Cardiac-specific overexpression of CIRBP was confirmed by immunoblot and immunofluorescence analysis of heart tissues (Figure 3F and Figure S2B). In line with previous findings 8, 19, ectopic augmentation of CIRBP appeared not to influence mouse cardiac function at the baseline level (Figure S2A) but evidently ameliorated DOX-induced cardiac dysfunction, as indicated by preservation of the EF and FS (Figure 3A). After DOX administration, there was a marked decrease in the HW/TL ratio and an overt increase in myocardial injury, both of which were attenuated by CIRBP overexpression, nevertheless (Figure 3B and C). Furthermore, cardiac apoptosis was evaluated in the different groups. We discovered that restoration of CIRBP expression in the myocardium successfully rescued DOX-induced cardiomyocyte apoptosis, as evidenced by reductions in TUNEL staining (Figure 3D and E) and cleaved caspase 3 levels (Figure 3F and G). Collectively, these results indicate that CIRBP attenuates DOX-induced cardiotoxicity in mice.

### Identification of CIRBP downstream effectors accounting for cardiomyocyte apoptosis

**Figure 4:**
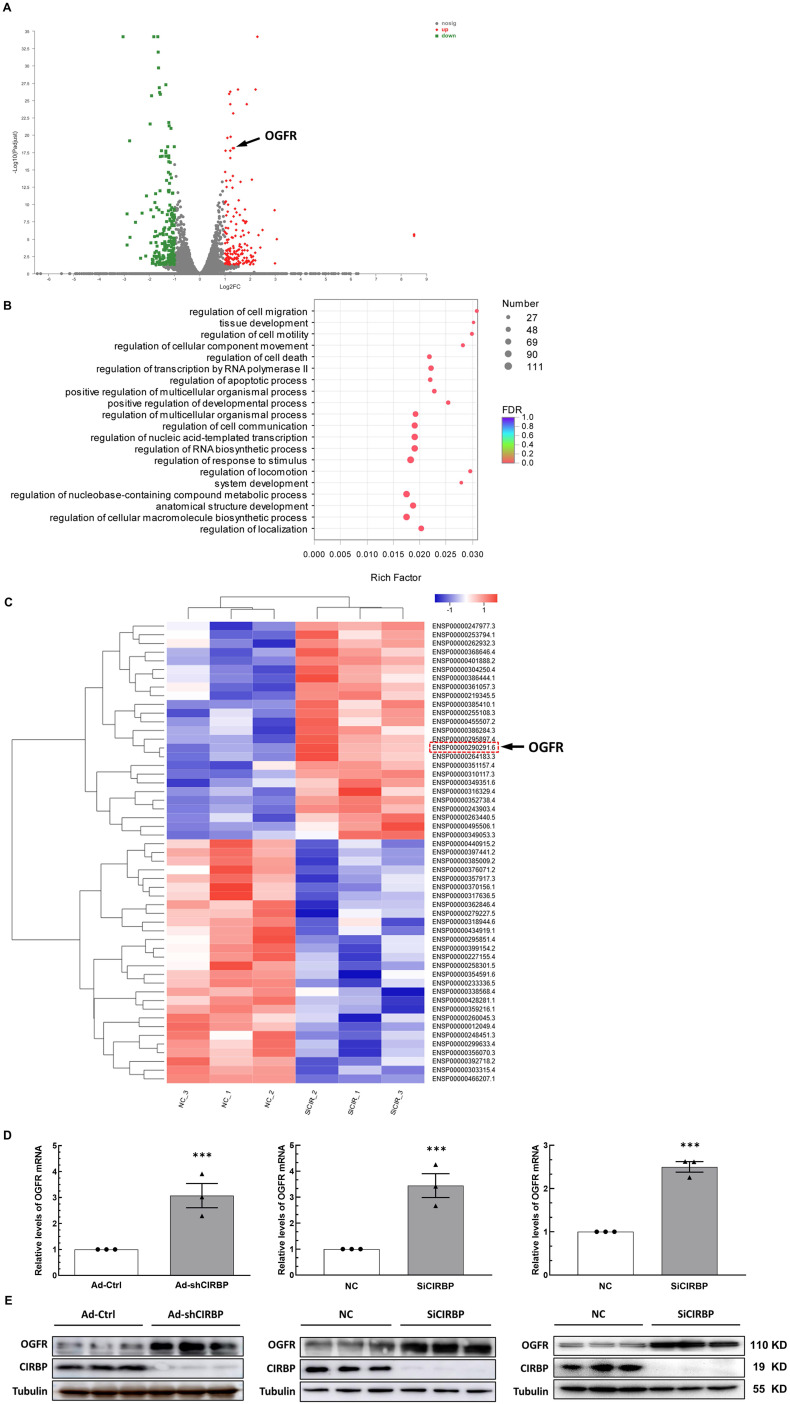
** Exploration of the downstream effector of CIRBP that likely accounts for cardiomyocyte apoptosis. A,** Volcano plot of differentially regulated transcripts in AC16 cells transfected with NC siRNA compared with those transfected with SiCIRBP. Opioid growth factor receptor (OGFR) is indicated by the arrow. **B,** Gene ontology (GO) enrichment analysis of the genes with altered expression between the NC siRNA- and SiCIRBP-transfected group, as determined by transcriptomics analysis. **C,** Heatmap showing differentially expressed proteins between the NC siRNA- and SiCIRBP-transfected groups identified by proteomics analysis. OGFR is indicated by the red dashed rectangle and indicated by the arrow. **D,** Relative levels of OGFR mRNA in human cardiomyocytes infected with an adenovirus vector expressing a control shRNA (Ad-Ctrl)- or CIRBP shRNA (Ad-shCIRBP)-expressing cassette or transfected with NC siRNA or SiCIRBP. Left, hiPSC-CMs; middle, AC16 cells; Right, T0519 cells. **E,** Immunoblot analysis of cellular proteins isolated from different cardiac myocytes described in D. The data are presented the mean ± SD of three independent experiments. Statistical significance was analysed using Student's *t*-test. ***, *p* < 0.001.

Previous studies have suggested that CIRBP defends cells from apoptosis most likely by activating certain anti-apoptotic signaling, such as the MAPK/ERK_1/2_ and NF-κB pathways 9-11, Of note, these pathways do not appear to be involved in DOX-induced cardiomyocyte apoptosis notwithstanding quenched CIRBP (Figure S3A), indicative of certain novel mechanisms mediating apoptosis associated with the loss of CIRBP. Identifying the latent effector accounting for DOX-induced cardiomyocyte apoptosis downstream of CIRBP thus becomes warranted. To this end, the combined transcriptomics and proteomics analysis between negative control siRNA (NC)- and CIRBP siRNA (SiCIRBP)-treated AC16 cardiac myocytes was performed. Correlation analysis and principal component analysis (PCA) of the RNA-seq data indicated discrepancies between triplicate NC siRNA- and SiCIRBP-treated samples (Figure S3B and C). Thus, samples NC_1 and SiCIRPB_2, which showed the most discrepancies, were excluded from the analysis. As shown in Figure 4A and Figure S3D, 171 mRNA transcripts in the SiCIRBP group were found to be upregulated and 216 ones were downregulated when comparing to the NC group (Table S1). Gene Ontology (GO) enrichment analysis of these altered transcripts further predicted a regulatory role for CIRBP in cell apoptosis (Figure 4B). Moreover, the iTRAQ quantitative proteomics data revealed that 53 proteins were significantly altered in expression upon CIRBP knockdown, with 25 increased and 28 decreased, respectively (Figures 4C, S3E and Table S2). Intriguingly, OGFR was highlighted as a downstream effector gene shared by both transcriptomics analysis and proteomics analysis. Furthermore, the ability of CIRBP to markedly repress OGFR expression in different human-derived cardiomyocytes was corroborated by real-time qPCR (Figure 4D; left, hiPSC-CMs; middle, AC16; right, T0519) and western blotting (Figure 4E; left, hiPSC-CMs; middle, AC16; right, T0519). Since OGFR is well documented to govern cellular homeostasis, which is related to proliferation and survival 12, 20, the CIRBP-OGFR regulatory axis most likely plays a fundamental role in handling with DOX-triggered cardiomyocyte apoptosis.

### CIRBP destabilizes OGFR mRNA via interacting with it

**Figure 5:**
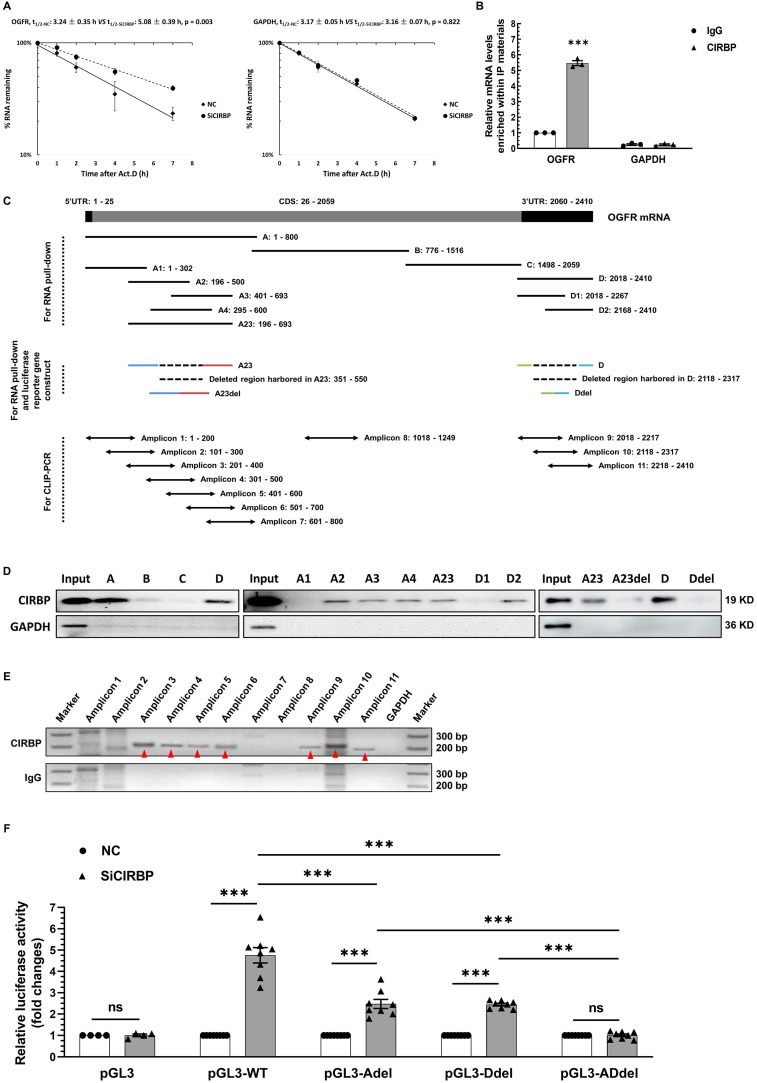
** CIRBP represses OGFR expression by destabilizing OGFR mRNA. A,** Analysis of the half-life (t_1/2_) of OGFR (left) and GAPDH (right) mRNA in AC16 cells transfected with NC siRNA or SiCIRBP after exposure to actinomycin D by real-time qPCR. **B,** RNA immunoprecipitation (RIP) with IgG and CIRBP antibodies using lysates prepared from AC16 cells. GAPDH mRNA served as a control. **C,** Schematic depiction of truncated fragments of OGFR mRNA used for RNA pull-down, crosslinked RNA immunoprecipitation coupled with reverse transcription polymerase chain reaction (CLIP-PCR), and the generation of luciferase reporter constructs. **D,** RNA pull-down analysis of the interactions between CIRBP and the different truncated RNA fragments shown in C. The protein lysates used for the pull-down assay were prepared from AC16 cells. GAPDH served as a control. **E,** Mapping of the CIRBP-binding regions in OGFR mRNA in AC16 cells through CLIP-PCR. IgG and GAPDH mRNA served as controls at different levels. The amplified specific bands are indicated by the red arrowheads. **F,** Measurement of luciferase activity in AC16 cells expressing different reporter constructs and transfected with NC siRNA or SiCIRBP. n=8 per group, except for PGL3 (n=4). The data are presented the mean ± SD of three independent experiments unless otherwise specified. Statistical significance was analysed by one-way ANOVA followed by Tukey-Kramer multiple comparisons. ***, *p* < 0.00; ns, no significance.

To elaborate on the level at which OGFR is regulated by CIRBP, we first determined whether the primary OGFR mRNA level is influenced upon CIRBP disruption. As displayed in Figure S4A, CIRBP knockdown had no impact on the primary OGFR mRNA level. Thus, we proposed that CIRBP most likely regulates the stability of OGFR mRNA. To provide further evidence for this hypothesis, the half-life of OGFR mRNA was analysed in AC16 cells. Our results suggested that the half-life of OGFR mRNA in CIRBP-silenced cells was markedly longer than that observed in NC siRNA-transfected cells (NC, 3.24 ± 0.35 h *vs.* SiCIRBP, 5.08 ± 0.39 h). Knockdown of CIRBP did not alter the half-life of GAPDH mRNA, which was assessed as a control (NC, 3.17 ± 0.05 h *vs.* SiCIRBP, 3.16 ± 0.07 h) (Figure 5A). CIRBP has been well characterized to act as an RNA-binding protein to regulate gene expression 7, Therefore, CIRBP-mediated destabilization of OGFR mRNA is thought to be attributed to the interaction between CIRBP and OGFR mRNA. Subsequently, RNA immunoprecipitation (RIP) was exploited to authenticate this association. OGFR mRNA was markedly enriched (~ 5.47-fold) in the protein fraction pulled down with CIRBP antibody compared to that pulled down with IgG, whereas GAPDH mRNA was not enriched in this fraction (Figure 5B).

To further investigate the region of OGFR mRNA that interacts with CIRBP, we conducted RNA pull-down and CLIP-PCR experiments. Biotinylated A, B, C and D fragments of OGFR mRNA and truncations of the A and D fragments, including A1, A2, A3, A4, A23, D1 and D2, were prepared and used for the pull-down assay (Figure 5C). The presence of CIRBP in the pulled down fractions was analysed by western blotting. As demonstrated in the left and middle panels of Figure 5D, the A, D, A2, A3, A4, A23, and D2 fragments interacted with CIRBP, but the B, C, A1, and D1 fragments did not. Moreover, the CLIP-PCR results showed that amplicons 3, 4, 5, 6, 9, 10, and 11 were amplified in the fractions pulled down with CIRBP antibody in the CLIP assay, whereas amplicons 1, 2, 7 and 8 were not detected. In sharp contrast, few non-specific bands were amplified in the IgG control group (Figure 5C and E). In keeping with pull-down results, CIRBP was found to bind predominantly to the regions covered by both the A23 fragment and D fragment. Additionally, an arbitrary deletion spanning the overlapping parts between the A2 and A3 fragments and the D1 and D2 fragments, which are depicted in Figure 5C, thoroughly abrogated the association of CIRBP with the A23 fragment and D fragment, respectively (Figure 5D right). These data unequivocally indicate that CIRBP mainly interacts with the two regions covered by the A23 and D fragments of OGFR mRNA, both the midsections of which likely serve as core interfaces for CIRBP binding.

Accordingly, a pGL3 reporter system carrying distinct combinations of inserts originating from OGFR mRNA (Figure S4B) was constructed to test whether CIRBP-induced inhibition of OGFR expression relies on the interaction between CIRBP and OGFR mRNA. Different chimeric reporter constructs were transfected into NC siRNA- and SiCIRBP-treated AC16 cardiomyocytes, and relative luciferase activity was assessed. As indicated in Figure 5F, CIRBP knockdown markedly increased luciferase activity in cells transfected with pGL3-WT (~ 4.76-fold), pGL3-Adel (~ 2.48-fold), and pGL3-Ddel (~ 2.44-fold) but failed to increase luciferase activity in cells transfected with either pGL3 (~ 1.01-fold) or pGL3-ADdel (~ 1.01-fold). Of note, an arresting superimposed increase in luciferase activity was observed in cells transfected with pGL3-WT compared with those transfected with pGL3-Adel and pGL3-Ddel, corroborating the existence of two CIRBP-binding regions in OGFR mRNA.

In aggregate, these findings suggest that CIRBP-mediated OGFR ablation depends mainly on the association of CIRBP with OGFR mRNA, which reduces mRNA stability and in turn decreases OGFR mRNA levels utilized for OGFR translation.

### CIRBP-mediated OGFR repression plays an essential role in averting cardiomyocytes from apoptosis induced by DOX

**Figure 6:**
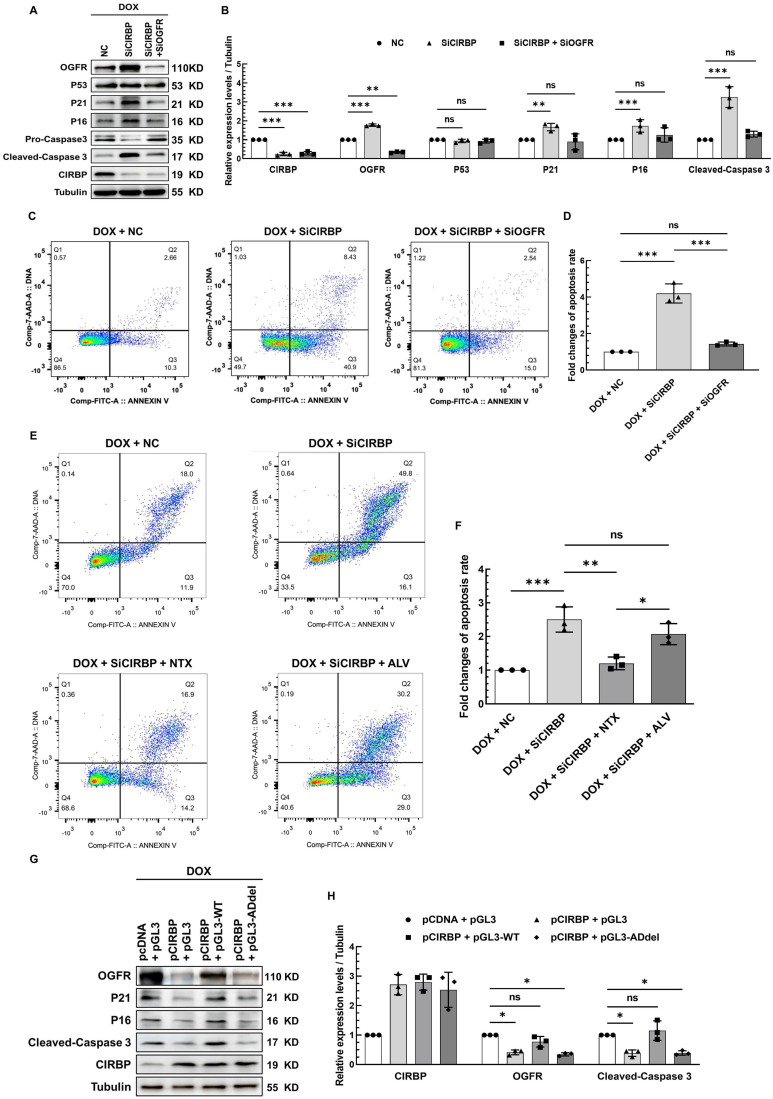
** CIRBP-mediated OGFR repression plays an essential role in protecting cardiomyocytes from apoptosis induced by DOX. A,** Representative immunoblots assessing DOX-induced apoptosis in AC16 cells transfected with NC siRNA, SiCIRBP, or siRNAs targeting both CIRBP and OGFR (SiCIRBP + SiOGFR). **B,** Densitometric quantification of the relative protein levels of CIRBP, OGFR, P53, P21, P16, and Cleaved-Caspase 3 in A. DOX-treated cells transfected with NC siRNA served as controls for quantification. **C,** Flow cytometry analysis of apoptosis of the cells described in A. **D,** Statistical analysis of the apoptosis rate in C. E, Flow cytometry analysis of DOX-induced apoptosis of AC16 cells treated with NC siRNA (DOX + NC), SiCIRBP (DOX + SiCIRBP), SiCIRBP plus naltrexone (NTX) (DOX + SiCIRBP + NTX), or SiCIRBP plus alvimopan (ALV) (DOX + SiCIRBP + ALV). **F,** Statistical analysis of the apoptosis rate in **E. G,** Representative immunoblots assessing DOX-induced apoptosis in AC16 cells co-transfected with pCDNA and pGL3 constructs (pCDNA + pGL3), pCIRBP and pGL3 constructs (pCIRBP + pGL3), pCIRBP and pGL3-WT constructs (pCIRBP + pGL3-WT), or pCIRBP and pGL3-ADdel constructs (pCIRBP + pGL3-ADdel). **H,** Densitometric quantification of the relative protein levels of CIRBP, OGFR, and Cleaved-Caspase 3 in G. DOX-treated cells cotransfected with pCDNA and pGL3 constructs served as controls for quantification. The quantitative data are presented as the mean ± SD of three independent experiments. Statistical significance was analysed by one-way ANOVA followed by Tukey-Kramer multiple comparisons. *, *p* < 0.05; **, *p* < 0.01; ***, *p* < 0.001; ns, no significant.

OGFR, a core component of the OGF/OGFR signaling, has been widely documented before to modulate cell survival, proliferation, senescence and apoptosis, wherein p21 and p16 have hitherto been proven to be two direct downstream effector genes responsible for OGF/OGFR signal transduction 21-23. Without exception, we found that OGFR expression was significantly induced in AC16 cardiomyocytes treated with all three chemotherapeutic drugs (Figure S5A), suggesting a central role for OGFR in cardiomyocyte apoptosis induced by various chemotherapies. To unveil the contribution of CIRBP-mediated OGFR suppression to this apoptotic process, multiple intervention strategies targeting OGFR, including CIRBP knockdown, administration of an OGFR blocker, and disruption of the interaction of CIRBP with OGFR mRNA, were adopted in cardiomyocytes exhibiting DOX-elicited apoptosis. In accord with the observations described above, CIRBP knockdown predisposed various types of cardiomyocytes to apoptosis, as evidenced by elevation of cleaved caspase 3 expression (AC16, Figure 6A and B; hiPSC-CMs, Figure S5B, left; T0519, Figure S5B, right) and the cellular apoptosis rate (AC16, Figure 6C and D), while simultaneous silencing of OGFR apparently abolished these apoptosis-related changes (Figures 6A-D and S5B) and attenuated the concomitant elicitation of p21 and p16 expression (Figure 6A and B) via the OGF/OGFR axis. Notably, the extensively studied p53-p21 pro-apoptotic signaling pathway appeared to not be involved in CIRBP deficiency-mediated susceptibility to apoptosis, as the expression of p53 was not altered in this experiment (Figures 6A, B and S5E).

Furthermore, Naltrexone (NTX), a commonly accepted treatment for drug and alcohol addiction as well as a potent antagonist of OGFR 13, 15, 24, successfully rescued the increase in apoptosis induced by CIRBP knockdown. To rule out that this effect was due to the latent action of NTX on opioid receptors 15, 16, another agent, Alvimopan (ALV), which has broad-spectrum inhibitory activity capable of pan-targeting multiple opioid receptors, including the δ, κ, and μ receptors 14, was employed as a control. Cardiomyocytes pre-treated with ALV showed little decrease in cell apoptosis (Figures 6E, F, S5C, and S5D). These results declared that OGFR activation, as manifested by changes in p21 and p16 expression (Figure S5E), is required for CIRBP deficiency-mediated exacerbation of DOX-induced cardiomyocyte apoptosis. Next, the foregoing construct pGL3-WT was utilized to competitively disrupt the interaction between CIRBP and OGFR mRNA. Transfection of AC16 cardiomyocytes with pGL3-WT, which showed a comparable transfection efficiency as pGL3-derived constructs (Figure S5F, luciferase CR), successfully disrupted the CIRBP overexpression-induced reduction in OGFR, p21, p16, and cleaved-caspase 3 expression, whereas the control construct pGL3 and pGL3-ADdel, in which the CIRBP-binding regions (A23 and D) are deleted, did not (Figures. 6G, H and S5F), further underscoring the importance of the association between CIRBP and OGFR mRNA in governing the cytoprotection of CIRBP against DOX-induced apoptosis. Based on these results, we conclude that the CIRBP-OGF/OGFR regulatory axis plays an essential role in modulating cardiomyocyte apoptosis during chemotherapy. Meddling in this signaling pathway, such as by altering OGFR expression and/or activation and disrupting the interaction between CIRBP and OGFR mRNA, seems to be a feasible strategy for regulating this pathological process.

### OGFR blockade ameliorates CIRBP ablation-aggravated myocardial apoptosis and cardiotoxicity during DOX administration

**Figure 7:**
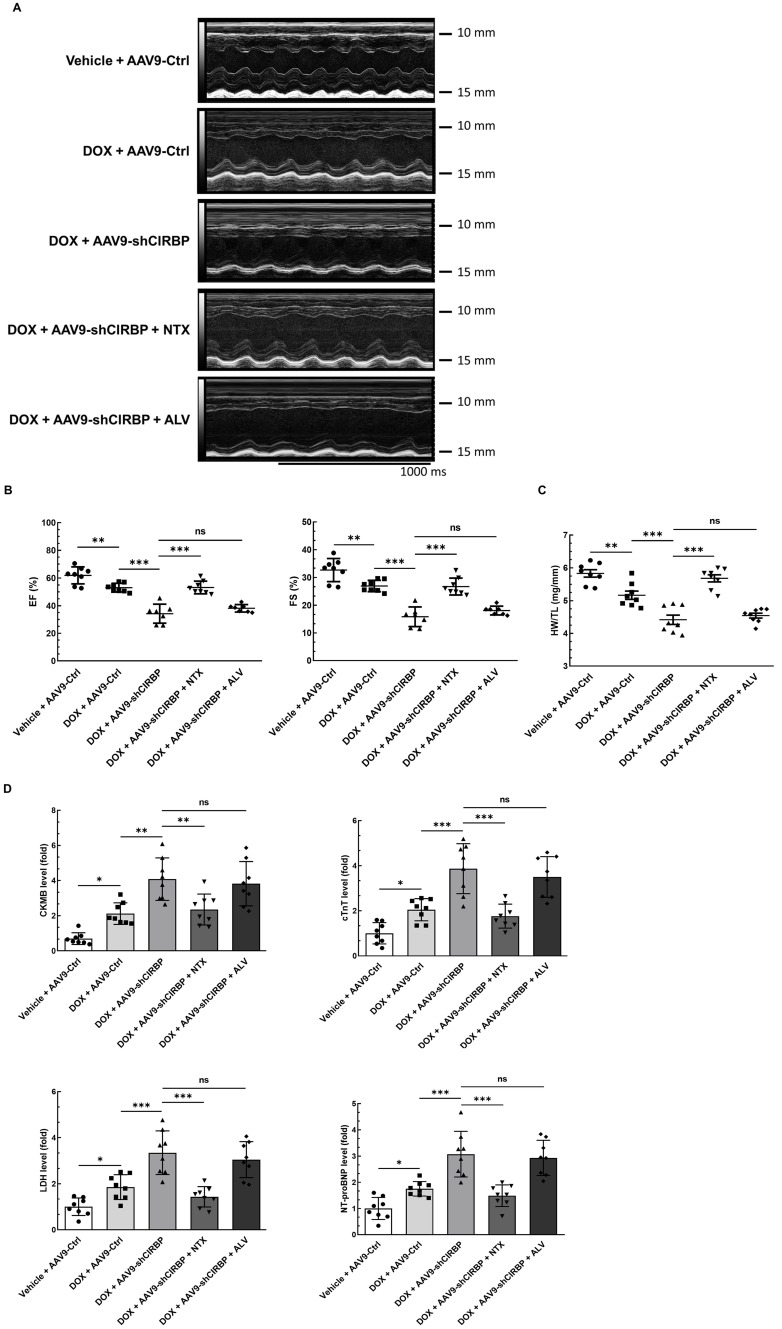

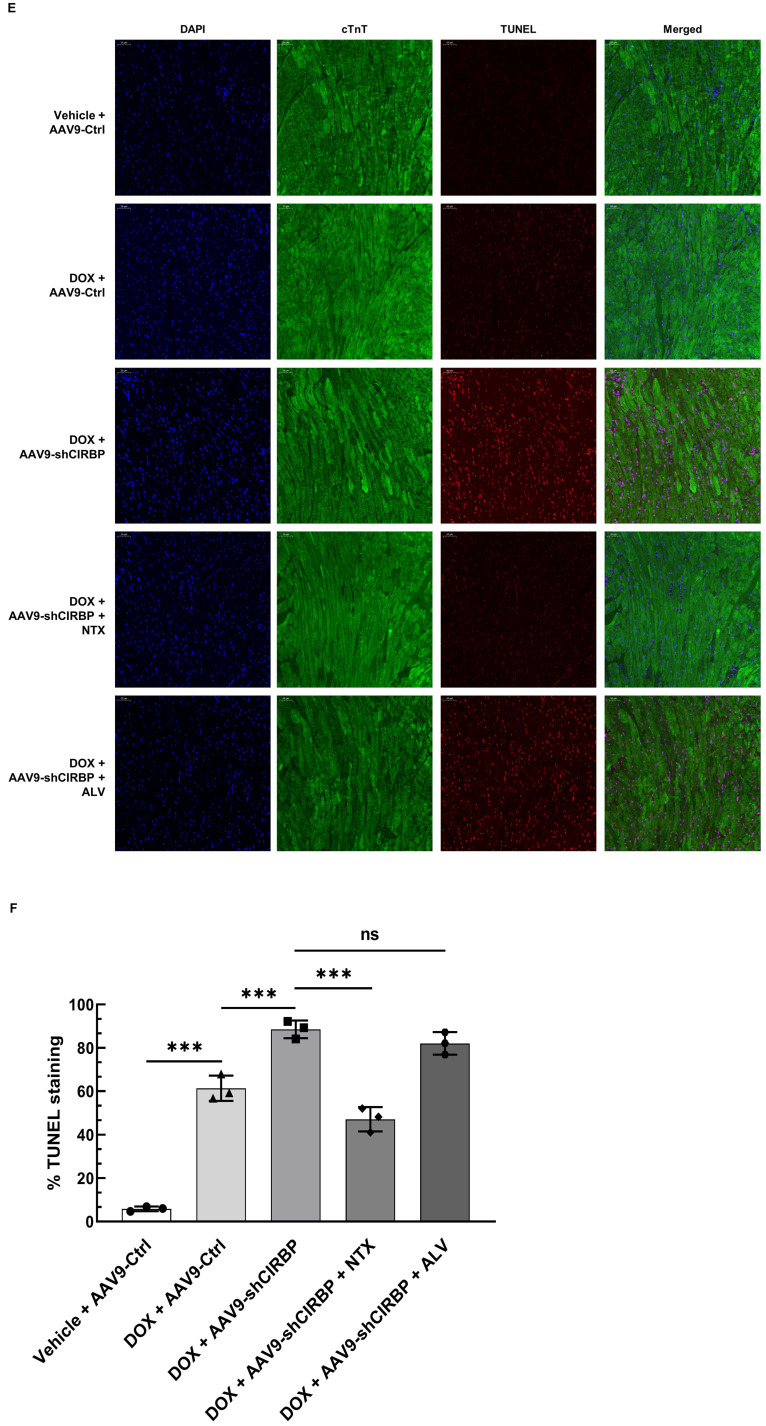
** OGFR blockade ameliorates CIRBP deficiency-aggravated myocardial apoptosis and cardiotoxicity during DOX administration. A,** Echocardiographic analysis of cardiac function in mice in the vehicle + AAV9-Ctrl groups (pre-injected with a control AAV9 vector and not treated with DOX), the DOX + AAV9-Ctrl group (pre-injected with control a AAV9 vector and treated with DOX), the DOX + AAV9-shCIRBP group (pre-injected with an AAV9 shCIRBP vector and treated with DOX), the DOX + AAV9-shCIRBP + NTX group (pre-injected with an AAV9 shCIRBP vector and NTX and treated with DOX), and the DOX + AAV9-shCIRBP + ALV group (pre-injected with an AAV9 shCIRBP vector and ALV and treated with DOX). Representative echocardiograms for each group. Vehicle + AAV9-Ctrl, n = 8; DOX + AAV9-Ctrl, n = 8; DOX + AAV9-shCIRBP, n = 7; DOX + AAV9-shCIRBP + NTX, n = 8; DOX + AAV9-shCIRBP + ALV, n = 7. Statistical analysis of EF, FS (**B**) and the HW/TL ratio (**C**) of mice in A. **D,** Biochemical determination of the relative serum levels of CK-MB, cTnT, LDH, and NT-proBNP for assessment of cardiac injury in the mice in A. **E,** Representative TUNEL staining images of heart tissue sections from the mice in A. Nuclei, DAPI (blue); cardiomyocytes, cTnT (green); TUNEL (red). Scale bar, 50 µm. **F,** Quantification of TUNEL staining in 3 microscopic fields from the hearts of the mice in A. The data are presented as the mean ± SD. Statistical significance was analysed by one-way ANOVA followed by Tukey-Kramer multiple comparisons. *, *p* < 0.05; **, *p* < 0.01; ***, *p* < 0.001; ns, no significance.

Despite CIRBP loss does not affect baseline cardiac mechanical function 8, 19, CIRBP indeed functions as an important cytoprotective factor when exposed to stresses threatening cellular survival 7, which is in accordance with our findings. As a proof of concept, we specifically dampened cardiac CIRBP expression via an AAV9 virus carrying a CIRBP shRNA-expressing cassette driven by the cTnT promoter to dissect the role of CIRBP-mediated repression of OGFR expression in dealing with DOX-induced cardiotoxicity. As anticipated, cardiac-specific knockdown of CIRBP (Figure S6A-C) significantly aggravated cardiotoxicity in mice receiving DOX treatment, as manifested by aberrant ventricular wall motion (Figure 7A), exacerbation of cardiac dysfunction (Figure 7B), an accelerated decrease in the HW/TL ratio (Figure 7C), and aggravation of cardiac injury (Figure 7D). The exacerbation of DOX-related toxicity in the mouse myocardium upon CIRBP ablation was accompanied by a dramatic increase in TUNEL staining of cardiomyocytes (Figure 7E and F) and elevation of cleaved caspase 3, OGFR, p21 and p16 expression (Figure S6B and C), indicative of a heightened apoptosis rate and activation of OGF/OGFR signaling in the mouse myocardium. However, intraperitoneal injection of NTX before DOX administration effectively ameliorated the apoptosis-associated cardiotoxicity in the CIRBP-deficient mouse heart, whereas ALV did not rescue this exacerbation of toxicity (Figures 7A to G, S6B and C). These results accentuate a central role of OGFR in arousing cardiac apoptosis under stress as well as an importance of CIRBP-mediated OGFR regulation in coping with cardiotoxicity, unambiguously shedding new light on cardioprotective interventions for cancer patients receiving chemotherapy.

## Discussion

In the present study, we reported that chemotherapy-induced cardiotoxicity involves myocardial CIRBP deficiency. Augmented expression of cardiac CIRBP significantly reduced DOX-induced cell apoptosis and heart injury and improved cardiac function, suggesting that CIRBP has a potent protective effect against chemotherapy-associated cardiotoxicity. To elaborate on this, combined transcriptomics and proteomics analysis of human immortalized cardiomyocyte AC16 deprived of CIRBP, mimicking CIRBP ablation upon exposure to chemotherapeutics, was carried out. OGFR was highlighted due to its tonic repressive role in cellular survival. CIRBP was discovered to destabilize OGFR mRNA by interacting with two interspaced regions harbored within OGFR mRNA, thus decreasing OGFR expression. CIRBP deficiency-rendered susceptibility of the myocardium to DOX-induced apoptosis and dyshomeostasis was found to depend primarily on OGFR expression and/or activation, as evidenced by the attenuated cardiac apoptosis and dysfunction when knocking down OGFR, sabotaging the association between CIRBP and OGFR mRNA with pGL3-WT-derived transcript, or paralyzing OGF/OGFR cascade with OGFR blocker Naltrexone. Altogether, these findings point out that CIRBP-OGFR regulatory axis plays a central role in the cardioprotection against chemotherapeutical insults.

CIRBP, initially found to be induced under cold shock, is now generally viewed as a stress-responsive protein affected by multiple physiological and pathological stimuli, including hypoxia, UV radiation, glucose deprivation, heat stress and circadian rhythm disruption 7, 25. Consistently, we reported that CIRBP expression is prominently reduced in cardiomyocytes exposed to chemotherapeutic agents such as DOX, cisplatin and 5-FU, further demonstrating the ability of CIRBP to handle with diverse stressors. Previous studies have documented the importance of CIRBP in maintaining cardiac function through modulation of cardiomyocyte repolarization 19 and hypothermic protection against oxidative stress and myocardial injury during cardiac surgery 8. In contrast, our findings suggest a critical role CIRBP plays in mitigating chemotherapy-related cardiotoxicity, stressing again the indispensability of CIRBP in sustaining cardiac homeostasis when coping with deleterious stimuli. CIRBP has been reported to prevent cell apoptosis via a myriad of pathways, including the p53, MAPK/ERK_1/2_ and NF-κB pathways 10, 26, 27, in different contexts and tissues and in the presence of various stimuli. Nevertheless, these proposed regulatory pathways appear not to explain the induction of cardiomyocyte apoptosis by chemotherapeutic insult, as the activity and expression of these mediators are not altered (Figures S3A, 6A, S5E and S6B). In addition, knockdown of CIRBP has been found to enhance the chemosensitivity of prostate cancer cells thus threatening cancer cell survival 28. However, the exact mechanism by which CIRBP acts on cell survival is unclear. Herein, we presented that CIRBP exerted the cytoprotective effect against chemotherapy-associated apoptosis through dampening OGF/OGFR growth inhibitory signal, which might be extrapolated to the observation that CIRBP disruption by heat shock renders prostate cancer cells susceptible to the chemotherapy.

CIRBP has been extensively investigated for its ability to modulate cellular redox status by promoting the expression of antioxidant genes, such as TRX and CoQs, to quench ROS, thereby benefiting cell survival 8, 29, 30. To our knowledge, ROS are reckoned as a culprit to arouse chemo-cardiotoxicity, resulting into crippled DNA repair system, disabled ATP synthesis, and cellular membrane damage 1, 4, 31. Given this, CIRBP-mediated ROS scavenging is conceived plausibly to be responsible for the cardioprotection of CIRBP against chemotherapy-produced detrimental alterations. This ROS hypothesis, however, has been tempered by a series of studies in which treatment with an ROS scavenger failed to prevent cardiac toxicity caused by DOX 4, 32. Beyond ROS, our study of CIRBP-mediated OGFR regulation undoubtedly provides an alternative explanation for cardiotoxicity induced by cancer therapy. Intriguingly, different opioid receptor antagonists have been reported to modulate cellular ROS levels 33-35. Hence, the relationship between CIRBP-mediated OGFR repression and ROS generation within cardiomyocytes needs further to be determined in the future to provide a comprehensive understanding of the role of CIRBP in cardioprotection.

OGFR is a non-canonical perinuclear opioid receptor that binds to native opioid ligands less efficiently than canonical opioid receptors. The pentapeptide OGF is currently the only well-characterized high-affinity endogenous ligand for OGFR. Data from previous studies suggest that OGF/OGFR signaling inhibits cell proliferation and survival during development, cancer, wound healing and angiogenesis 12. Notwithstanding the controversy whether ignition of the OGF/OGFR pathway alone provokes cell apoptosis 13, 21, 22, 36, 37, our results strongly suggest that exacerbation of DOX-induced cardiomyocyte apoptosis due to CIRBP ablation is attributed bona fide to OGFR upregulation and activation. Additionally, concomitant alterations in the expression of both p21 and p16, two well-characterized downstream effectors of OGF/OGFR in mediating tonic inhibition of growth and survival 21, 22, were observed in our study, further corroborating the contribution of OGFR to cardiomyocyte apoptosis. We therefore conclude that the aberrant activation of OGF/OGFR signaling triggered by CIRBP loss renders cardiomyocytes more susceptible to chemotherapy-induced apoptosis, thereby compromising the chemoresistance of the myocardium. Three strategies for disrupting CIRBP-OGF/OGFR signaling and thus mitigating cardiotoxicity, including decreasing OGFR expression via RNAi, competitively inhibiting the binding of CIRBP to OGFR mRNA, and inactivating OGFR with potent blockers, were suggested in this study. Given the inducibility of CIRBP, the ability of other strategies that elicit CIRBP expression during chemotherapy, such as mild hypothermia, hypoxic preconditioning, and time-window option for therapy at daily peak levels of circadian CIRBP, to reduce myocardial toxicity should be assessed. Needless to say, our work provides new insight into cardioprotection in patients receiving cancer therapy.

Despite the fruitful results obtained above, there are still several limitations in our study. CIRBP was demonstrated to be reduced in cardiomyocyte apoptosis evoked by agents with distinguishing potency in chemotherapy; however, the mechanisms underlying this reduction and whether they are different in response to different chemotherapeutic drugs are unknown, both of which require much more efforts to address. The myocardium comprises various types of cells, including cardiomyocytes, fibroblasts, pericytes, endothelial cells, smooth muscle cells, and immune cells. Thus, we should recapitulate chemotherapy-induced cardiotoxicity in distinct cellular context. Our study focused on the cardiomyocytes, virtually ignoring the contribution of apoptosis of other cardiac cells to the cardiotoxicity. Whether CIRBP-OGFR repressive regulation in other types of cells are halted simultaneously after treatment with chemotherapeutic agents, thereby cooperatively eliciting massive apoptosis in the myocardium, should be further investigated. In regard to the CIRBP-OGFR regulatory axis, we actually have not touched the more sophisticated mechanisms in charge of provoking cardiomyocyte apoptosis, although we did observe that the upregualtion of p21 and p16. Therefore, more detailed work needs to be performed to clarify the role of CIRBP-mediated repression of OGFR expression in cardioprotection against chemotherapeutic insult. Collectively, our findings delineate that chemotherapy-associated CIRBP deficiency paralyzed the association of CIRBP with OGFR mRNA, thus enhancing OGFR mRNA stability and aggrandizing cardiac OGFR levels, which considerably facilitates inhibitory OGF/OGFR signaling. Blocking OGFR with NTX potently disrupts OGF/OGFR signal transduction and dramatically attenuates cardiac apoptosis and toxicity (Graphical Abstract). These findings shed new light on the adoption of cardioprotective regimens during chemotherapy in the future.

## Supplementary Material

Supplementary materials and methods, figures, table legends.

Supplementary table 1.

Supplementary table 2.
