# Supplementary material for: CIRBP-OGFR axis safeguards against cardiomyocyte apoptosis and cardiotoxicity induced by chemotherapy
Source: Int J Biol Sci. 2022 Apr 11;18(7):2882–97. doi: 10.7150/ijbs.69655 (PMC9066098; doi:10.7150/ijbs.69655)
Supplement: Supplementary file 1 — Supplementary materials and methods, figures, table legends. [file ijbsv18p2882s1.pdf]

Supplementary materials

CIRBP-OGFR axis safeguards against cardiomyocyte apoptosis and cardiotoxicity induced by chemotherapy

Cihang Liu, Xiaolei Cheng, Junyue Xing, Jun Li, Zhen Li, Dongdong Jian, Ying Wang, Shixing Wang, Ran Li, Wanjun Zhang, Dongxing Shao, Xiaohan Ma, Xiru Chen, Jia Shen, Chao Shi, Zhiping Guo, Wengong Wang, Taibing Fan, Lin Liu, Hao Tang

Supplementary Materials and Methods

Constructs

To construct chimeric pGL3 reporters, A23 (196 - 693), A23del (deleted region, 351 - 550), D (2018 - 2410), and Ddel (deleted region, 2118 - 2317) fragments were de novo synthesized (Generay, Shanghai, China). The A23 and A23del fragments were inserted between the Nco I and Hind III sites of the pGL3-promoter vector (Promega), whereas the D and Ddel fragments were inserted at the Xba I site. To generate a CIRBP overexpression construct, the human CIRBP CDS fragment was amplified by PCR using the following primers: GCAAGCTTATGGCATCAGATGAAGGCAAAC (forward) and GCGGATCCTTACTCGTTGTGTGTAGCTGAGGA (reverse). The fragment was then inserted between the Hind III and BamH I sites of the pcDNA^{TM}3.1(+) vector (Invitrogen). To construct pcDNA3.1(+)-OGFR for generating different templates for *in vitro* transcription, full-length OGFR mRNA was de novo synthesized (Generay, Shanghai, China). Then, the synthesized fragment was inserted between the Hind III and Not I sites of the pcDNA^{TM}3.1(+) vector (Invitrogen).

Cell culture and treatments

AC16 cardiomyocytes were cultured in Hyclone high-glucose DMEM supplemented with 10% FBS. The immortalized

ventricular cardiomyocyte T0519 was purchased from Abm Corporation (Canada) and cultured in Prigrow I medium (Cat. No. TM001) supplemented with 10% foetal bovine serum and 1% antibiotics at 37 °C in 5% CO2. Human iPSC-derived cardiomyocytes (hiPSC-CMs), well differentiated (40 days) and tested in purity and electrophysiological characteristics of the cardiomyocytes, were purchased from Help Stem Cell Innovations Co., Ltd. (Nanjing, China) and maintained in culture following the manufacturer’s instruction.

Neonatal rat ventricular myocytes (NRVMs) from 1- to 3-day-old Sprague-Dawley rats were isolated and maintained according to a previously described standard protocol.(1) When these cells reached 50% confluence, they were treated with chemotherapeutics (doxorubicin (DOX), cisplatin, and 5-fluorouracil (5-FU)) (MedChemExpress) respectively, and collected for analysis 24 h later. For OGF/OGFR axis inhibition, AC16 cells were pretreated with NTX (300 nM, purchased from Selleck) or ALV (300 nM, purchased from Selleck) for 2.5 h.(2) All plasmid transfections were performed using Lipofectamine 2000 (Invitrogen). Unless otherwise indicated, the cells were analysed 48 h after transfection. To silence CIRBP, AC16 cells, T0519 cells or NRVMs were transfected with an siRNA targeting human CIRBP (GGCUCCAGAGACUACUAUA) or rat CIRBP (AUUUUCAAAGGUGACAAACCC) by using Lipofectamine RNAiMAX (Invitrogen), and hiPSC-CMs were infected with recombinant adenovirus (rAdV) carrying CIRBP shRNA expression cassette (Hanbio Biotechnology Co., Shanghai, China). To silence OGFR, AC16 or T0519 cells were transfected with an siRNA targeting human OGFR (UAGAAACUCAGGUUUGGCG) by using Lipofectamine RNAiMAX (Invitrogen), and hiPSC-CMs were infected with recombinant adenovirus (rAdV) carrying OGFR shRNA expression cassette (Hanbio Biotechnology Co., Shanghai, China).

Mouse model and treatments

C57BL/6 male mice (6-7 weeks old) were purchased from the Institute of Laboratory Animal Science, Chinese Academy of Medical Sciences (Beijing, China) and were subjected to adaptive feeding for 1 week before the study commenced. All

mice were maintained under specific pathogen-free, environmentally controlled (temperature: 20 - 25 °C; humidity: 50 ± 5%) barrier conditions in individual ventilated cages and were fed sterile food and water ad libitum. To specifically overexpress or knockdown CIRBP in the myocardium, mice were given a single intravenous injection of an AAV9 vector expressing mouse CIRBP (AAV9-CIRBP) or shCIRBP (AAAAGCUUGCCUUCAUCUGAU) (AAV9-shCIRBP) under the control of the cardiac troponin T (cTnT) promoter or an AAV9 vector expressing a negative control (AAV9-NC) via the tail vein at a concentration of 1×10^{11} viral genomes per mouse. The efficiency of virus infection and the expression of CIRBP in mouse cardiomyocytes were further assessed by immunofluorescence staining of GFP and western blot analysis. AAV9-CIRBP, AAV9-shCIRBP and AAV9-NC were generated by Hanbio Biotechnology Co. (Shanghai, China). Four weeks post-AAV9 injection, the mice were injected via the tail vein with DOX (5 mg/kg) once weekly for 4 weeks to generate a preclinical model of chemotheray-induced cardiotoxicity as described in previous studies.(3, 4) For NTX or ALV treatment, the mice were given intraperitoneal injection daily with either 15 mg/kg NTX, 15 mg/kg ALV, or 0.2 ml sterile saline (vehicle) once DOX administration. The mice were housed 5 or 6 per cage, and all mice in one cage belonged to the same treatment group. The mice were observed daily and weighed per week, and after 8 days of DOX treatment, they were subjected to echocardiography before being sacrificed. Mice were sacrificed under the isoflurane inhalation (1.4%) and followed by cervical dislocation

Western blotting

Western blot analysis was performed following standard procedures. Proteins were extracted with radioimmunoprecipitation assay (RIPA) buffer (25 mM Tris–HCl (pH 7.6), 150 mmol/L NaCl, 1% NP-40, 1% sodium deoxycholate, 0.1% SDS, and protease inhibitor cocktail). After homogenization, the samples were sonicated and centrifuged at 4 °C. The supernatants were transferred to fresh tubes, and the protein concentrations were determined using the bicinchoninic acid (BCA) method. Equal amounts of protein were separated by SDS-PAGE and transferred

onto polyvinylidene difluoride membranes (Millipore). After being blocked, the membranes were incubated with the following primary antibodies: OGFR (1:1000), Tubulin (1:2000), Pro-Caspase3 (1:1000), CIRBP (1:2000) (all purchased from Proteintech), Cleaved-Caspase3 (1:1000), NF-κB (1:1000), phospho-NF-κB (1:1000), ERK (1:1000), phospho-ERK (1:1000) (all purchased from CST), p16 (1:1000) (purchased from Abcam), p21 (1:1000) (purchased from Millipore), and p53 (1:2000) (purchased from Santa Cruz Biotechnology). After the membranes were washed and incubated with the appropriate horseradish peroxidase-conjugated secondary antibody (Santa Cruz Biotechnology), the immune complexes were visualized with chemiluminescence reagent and detected with an AI800 instrument (GE).

Real-time qPCR

RNA was extracted from materials with TRIzol® Reagent according to the manufacturer's instructions (Invitrogen) and treated with *TransScript*® II Reverse Transcriptase for cDNA synthesis (Transgen, Beijing, China). A two-step real-time qPCR amplification reaction was performed by using ChamQ Universal SYBR qPCR Master Mix (Vazyme, Nanjing, China) and a Bio-Rad CFX96TM Real-time System. To quantify the levels of different mRNAs, RT-qPCR analysis was carried out using the following primer pairs: ACTACTATAGCAGCCGGAGTC and TCGGAGTGTGACTTACCGT for human CIRBP mRNA, CCCGAGAGGTCTTTTTCCGAG and CCAGCCCATGATGGTTCTGAT for human BAX mRNA, GGTGGGGTCATGTGTGTGG and CGGTTCAGGTACTCAGTCATCC for human BCL2 mRNA, CTGGGCTACACTGAGCACC and AAGTGGTCGTTGAGGGCAATG for human GAPDH mRNA, GAACGAGACTGCAATGGGGA and CAATGAAACAGCCGTTGGGC for human OGFR mRNA, CTGCGAGAACCAGGAGTGAAC and CACCAAATGCCTACTGCCAG for human OGFR primary mRNA, AAGATGGAACCGCTGGAGAG and CGTAAGTGATGTCCACCTCG for luciferase mRNA, CTTCAGAACTGGACGGACA and TGGTTCTCGCAGAGGAAA for Adel, CCTCAGGCTCTGCTTCGT and GGCTTCTGGTGGCTGCTA for Ddel.

RNA sequencing (RNA-seq)

Total RNA was extracted from tissue using TRIzol® reagent according to the manufacturer's instructions (Invitrogen), and genomic DNA was removed using DNase I (Takara). Then, RNA quality was determined with a 2100 Bioanalyser (Agilent) and quantified using an ND-2000 spectrophotometer (NanoDrop Technologies). Only high-quality RNA samples (OD260/280 = 1.8~2.2, OD260/230 ≥ 2.0, RIN ≥ 6.5, 28S:18S ≥ 1.0, > 1 μg) were used to construct a sequencing library. An RNA-seq transcriptome library was prepared using a TruSeqTM RNA Sample Preparation Kit from Illumina (San Diego, CA) and 1 μg of total RNA. After quantification with a TBS380 instrument, the paired-end RNA-seq sequencing library was sequenced with an Illumina HiSeq xten/NovaSeq 6000 sequencer (2 ×150 bp read length). Differential expression analysis was performed using DESeq2, DEGseq, and EdgeR with a Q value ≤ 0.05 (genes with a |log2FC| > 1 and Q value ≤ 0.05 according to DESeq2 and EdgeR and a Q value ≤ 0.001 according to DEGseq were considered significantly differentially expressed genes (DEGs)).

iTRAQ quantitative proteomics

The samples were suspended in protein lysis buffer (8 M urea, 1% SDS) containing an appropriate protease inhibitor to inhibit protease activity. Then, the mixture was ultrasonicated at 40 kHz and 40 W for 2 min and then incubated on ice for 30 min. After centrifugation at 12000 × g and 4 °C for 30 min, the concentration of the protein supernatant was determined by the BCA method with a BCA Protein Assay Kit (Thermo Scientific). Protein quantification was performed according to the protocol of the kit, and then analysis was performed using standard procedures. Briefly, the peptides were labelled with iTRAQ tags (Applied Biosystems) and analysed by on-line electrospray tandem mass spectrometry on a Nano Aquity UPLC system (Waters Corporation) connected to an LTQ Orbitrap XL mass spectrometer (Thermo Scientific) equipped with an on-line nano electrospray ion source (Michrom Bioresources). Proteins identified by two or more unique peptides were selected for further analysis. Differentially expressed proteins were selected according to the

following criteria: (1) an adjusted p value < 0.05 and (2) a fold change < 0.83 or fold change > 1.2.

Analysis of mRNA stability

To measure the half-life of endogenous OGFR mRNA, actinomycin D (2 μg/ml) was added to the cell culture medium, and total RNA was isolated at the indicated times and subjected to RT-qPCR analysis using OGFR-specific primers. GAPDH mRNA was used as a negative control (NC).

RNA immunoprecipitation (RIP)

For crosslinking of ribonucleoprotein (RNP) IP complexes, cells were exposed to UVC (254 nm, 400 mJ/cm^2), and whole-cell lysates were prepared for immunoprecipitation using an anti-CIRBP antibody. Briefly, the lysates were precleaned (30 min, 4 °C) by using 3 μg of IgG and 20 μl of Protein G Dynabeads (Thermo Scientific) that had been previously soaked in NT2 buffer (50 mM Tris, pH 7.4, 150 mM NaCl, 1 mM $MgCl_2$, and 0.05% Nonidet P-40) supplemented with 5% BSA. The lysates (300 μg) were incubated with 20 μl of Protein G Dynabeads (Thermo Scientific) in the presence of 3 μg of antibody for 3 h at 4 °C. The IP materials were washed twice with stringent buffer (100 mM Tris-HCI (pH 7.4), 500 mM LiCl, 0.1% Triton X-100, 1 mM dithiothreitol (DTT), 2 μg/ml leupeptin, 2 μg/ml aprotinin, and 1 mM phenylmethylsulfonyl fluoride) and twice with IP buffer. The transcripts present in the RNP complexes were analysed by RT-qPCR.

RNA pull-down

pcDNA3.1-OGFR was used as a template for PCR amplification of the different OGFR mRNA fragments. To prepare templates for the A, B, C, D, A1, A2, A3, A4, A23, D1, D2 fragments, the following primer pairs were used: (T7) AGCGCGAGCCCCGCC and ACATGAAGTAGTCCAGGGCACTCTG for fragment A, (T7)CAGAGTGCCCTGGAC

and CTCACTGTGTCCAGCCTTGGGGTGC for fragment B, (T7)CAAGGCTGGACACAG and TTAAGGCTTCCCAGACTTGGCAGAAG, (T7)CAAGGCTGGACACAG and TTAAGGCTTCCCAGACTTGGCAGAAG for fragment C, (T7)CGCAGAGGTGGAGTC and CAAAGTCAAATGAATTTATTCAGAAAAGGC for fragment D, (T7)AGCGCGAGCCCCGCC and TTGGCGTGTCCCCATTGCAG for fragment A1, (T7)GTCCAGAATGACAGGGTCCAGA and CTTTTAAACACCTCGACCTCCCTGAG for fragment A2, (T7)GACAATCACTCCTACATCCAGTG and AGCTCACCCAGCGACTTGAGGAT for fragment A3, (T7)CACGCCAAACCTGAGTTTCTACAG and TATTACGGCCCACCGTGCCCGT for fragment A4, (T7)GTCCAGAATGACAGGGTCCAGA and AGCTCACCCAGCGACTTGAGGAT for fragment A23, (T7)CGCAGAGGTGGAGTC and AGGCCAGGAGGCTTCTGCA for fragment D1, (T7)CATGACCCACAGTGCTGGC and CAAAGTCAAATGAATTTATTCAGAAAAGGC for fragment D2, (T7)GTCCAGAATGACAGGGTCCAGA and AACAGCCGTTGGGCAGGAAG for fragment A23-1, (T7)TCATTGAGGACATTC and CAGCATGAGCTCGTAGGCCCG for fragment A23-2, and (T7)GGCTTCTACGGGATCC and AGCTCACCCAGCGACTTGAGGAT and fragment A23-3.

For biotin pull-down assays, PCR-amplified DNA was used as a template to transcribe biotinylated RNA by using T7 RNA polymerase in the presence of biotin-UTP. One microgram of purified biotinylated transcripts was incubated with 100 μg of cytoplasmic extracts for 30 min at room temperature. The complexes were isolated with paramagnetic streptavidin-conjugated Dynabeads (Invitrogen), and the pulled down material was analysed by western blotting.

Crosslinked RNA immunoprecipitation coupled with reverse transcription polymerase chain reaction (CLIP-PCR)

Crosslinked RNA immunoprecipitation (CLIP) was performed following a previously reported protocol.(5) Briefly, cells were exposed to UVC at 254 nm (400 mJ/cm^2), and whole-cell lysates were subsequently prepared with NP-40 lysis

buffer supplemented with protease inhibitor and 1 mM DTT. The supernatants were collected after centrifugation, and RNase T1 (New England Biolabs) was added to 1 U/µl final concentration for partial digestion of RNA in the 100 - 300 nt range. The prepared lysates (~ 1 ml) were incubated (16 h, 4 °C) with 20 µl of Protein G Dynabeads (Thermo Scientific) precoated with IgG or CIRBP antibody. The beads were washed at least 3 times with NP-40 lysis buffer and then incubated with 20 U of RNase-free DNase I (Genstar, Beijing, China) in 100 µl NP-40 lysis buffer for 15 min at 37 °C. A total of 700 µl of NP-40 lysis buffer was added, and the beads were incubated with 0.1% SDS and 0.5 mg/ml proteinase K (Sigma Aldrich) for 15 min at 55 °C. RNA isolation and purification were conducted with acidic phenol following standard procedures. The RNA was further treated with *TransScript*® II Reverse Transcriptase (Transgen, Beijing, China) for cDNA synthesis, and then amplicons 1 - 9 of OGFR mRNA were amplified by PCR using the following primer pairs: TTAGTAGCGCGAGCCCCGCC and TGGACTGGAACGAGCTGGGC, ACTGCGAGGACGGCGAGGCC and TTGGCGTGTCCCCATTGCAG, GTCCAGAATGACAGGGTCCAGA and GTCCTCAAGGAGGTCATAGTTGTCC, CACGCCAAACCTGAGTTTCTACAG and CTTTTAAACACCTCGACCTCCCTGAG, GAGGACAATCACTCCTACATCCAGTG and TATTACGGCCCACCGTGCCCGT, AGCTCCCAGGAGATCCAGGAG and AGCTCACCCAGCGACTTGAGGAT, CGAGCACAGAACTACCAGAAGCG and ACATGAAGTAGTCCAGGGCACTCT, GCCAGAGCATAGCAAGGGT and CTCCACCTCTGAGGCACTCTG, GACGCAGAGGTGGAGTCTTCTG and CTTCTGGTGGCTGCTATAGTGGC, ATATCCGGAGCTGCTGCGGGCT and GTCAATTTACAAAGACAGGGGCGCA, and TATAGCCGCGAGGCCCTCAGG and TCAAAGTCAAATGAATTTATTCAGAAAAGGC.

Dual-luciferase reporter assay

Cultured AC16 cells were transfected with reporters using Lipofectamine 2000 (Invitrogen). The cells were cotransfected with the PGL3 promoter vector as an internal control. Firefly and Renilla luciferase activities were measured with a

double luciferase assay system (Promega) following the manufacturer’s instructions. Firefly luciferase activity was normalized to Renilla luciferase activity in the same sample.

Immunofluorescence staining

Paraffin-embedded sections (3 μm) were deparaffinized in xylene, rehydrated by washing in ethanol and rinsed in PBS. Antigen retrieval was performed by pressure cooking the sections for 6 min in citrate buffer (pH 6.0, target retrieval solution, Dako, Santa Clara, CA, USA), and then the sections were blocked with 2.5% BSA. The sections were stained overnight at 4 °C with the following primary antibodies: rabbit anti-CIRBP, rabbit anti-cleaved caspase 3, and mouse anti-cTnT. After three washing steps, the sections were incubated with secondary antibodies conjugated to DyLight Fluor 488 and DyLight Fluor 550 for 1 h at room temperature, the nuclei were stained with 4’,6-diamidine-2’-phenylindole dihydrochloride (DAPI, Sigma Aldrich), and the slides were mounted with Permafluor mounting medium (Thermo Scientific). Images were taken on a Zeiss Axio observer Z1 microscope using Tissue FAXS software (version 6.06.245.103, Tissue Gnostics, Vienna, Austria). All histologic examinations were performed by an independent observer blinded to the treatment groups and time points.

Evaluation of apoptosis in cardiomyocytes and tissue sections

For flow cytometry, AC16 cells were grown overnight in 10 cm plates and treated with DOX for 24 h. Following treatment, the cells were trypsinized, pelleted, and resuspended in 100 μl of 1X Annexin V Binding Buffer. Then, 5 μl of FITC-conjugated Annexin V antibody and 5 μl of 7-amino-actinomycin D (7-AAD) were added. The cells were incubated in the dark at room temperature for 15 min, and then 400 μl of 1 × Annexin V Binding Buffer was added. The samples were analysed by an LSRFortessa flow cytometer (BD Biosciences) in one hour.

For TUNEL staining, tissues sections were dewaxed, hydrated and subjected to antigen retrieval. TUNEL staining was

performed using an in situ cell death detection kit (no. 11684795910, Roche) according to the manufacturer’s protocol. The apoptotic index was measured by counting TUNEL-positive puncta in 100 randomly selected actinin-positive cells in multiple randomly chosen fields at 400× magnification. Images were captured with a laser-scanning confocal microscope (SP8, Leica).

Echocardiographic assessment

Echocardiograms were recorded from conscious, gently restrained animals using a Vevo 3100 system with an MS400C scanhead. M-mode recordings were obtained at the level of the papillary muscles to measure systolic and diastolic left ventricular internal dimensions. Ejection fraction (EF) and fractional shortening (FS) were calculated as (LVIDd-LVIDs)/LVIDd and are expressed as percentages.

Biochemical determination

Mouse serum was collected, and the concentrations of cTnT, lactate dehydrogenase (LDH), creatine kinase-myocardial band (CK-MB) and N-terminal pro-B type natriuretic peptide (NT-proBNP) were measured using commercially available ELISA kits according to the manufacturer’s instructions (cTnT, no. E-EL-M1801c, Elabscience; D-LDH, no. E-EL-M0419c, Elabscience; CKMB, no. E-EL-M0355c, Elabscience; NT-proBNP, no. E-EL-M0834c, Elabscience.).

Supplementary figures and figure legends

Supplementary Figure 1

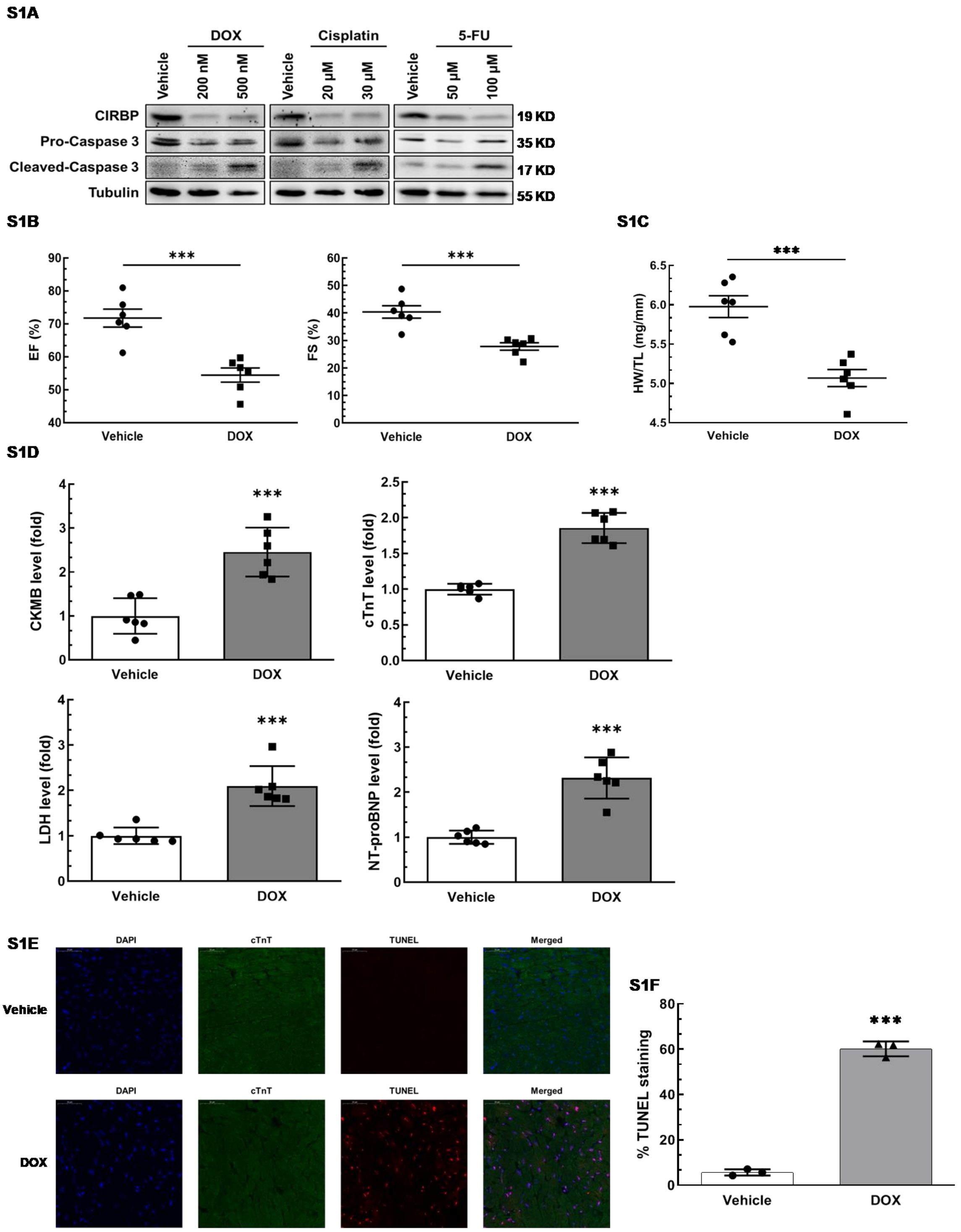

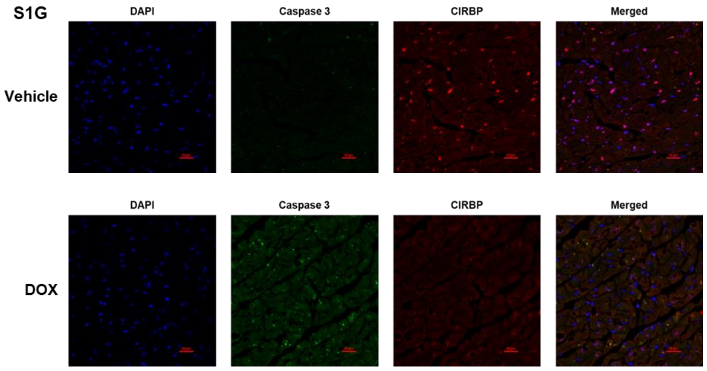

SFigure 1 Characterization of chemotherapy-induced cardiomyocyte apoptosis and cardiotoxicity

S1A, Representative immunoblots assessing apoptosis induced by three typical chemotherapeutics (doxorubicin (DOX), cisplatin, and 5-fluorouracil (5-FU)) in immortalized human ventricular cardiomyocytes (T0519). **S1B**, Echocardiographic evaluation of the cardiac function of mice administered vehicle or DOX. Left, ejection fraction (EF); right, fractional shortening (FS). n = 6 per group. **S1C**, Statistical analysis of the heart weight/tibia length (HW/TL) ratio of the mice in S1B. n = 6 per group. **S1D**, Biochemical determination of the relative serum levels of creatine kinase-myocardial band (CK-MB), cardiac troponin T (cTnT), lactate dehydrogenase (LDH), and N-terminal pro-B type natriuretic peptide (NT-proBNP) for assessment of cardiac injury in the mice in S1B. n = 6 per group. **S1E**, Representative TUNEL staining images of heart tissue sections from the mice in S1B. Nuclei, DAPI (blue); cardiomyocytes, cTnT (green); TUNEL (red). Scale bar, 50 μm. **S1F**, Quantification of TUNEL staining in 3 microscopic fields from the hearts of the mice in S1B. Vehicle-treated cells transfected with NC served as controls for quantification, and that data are presented as the mean ± SD of 3 independent experiments unless otherwise specified. **S1G**, Representative immunofluorescence images of heart tissue sections from the mice in S1B. Nuclei, DAPI (blue); Cleaved-Caspase 3 (green); CIRBP (red). Scale bar, 20 μm. Statistical significance was analysed using Student’s t-test. ***, $p < 0.001$.

Supplementary Figure 2

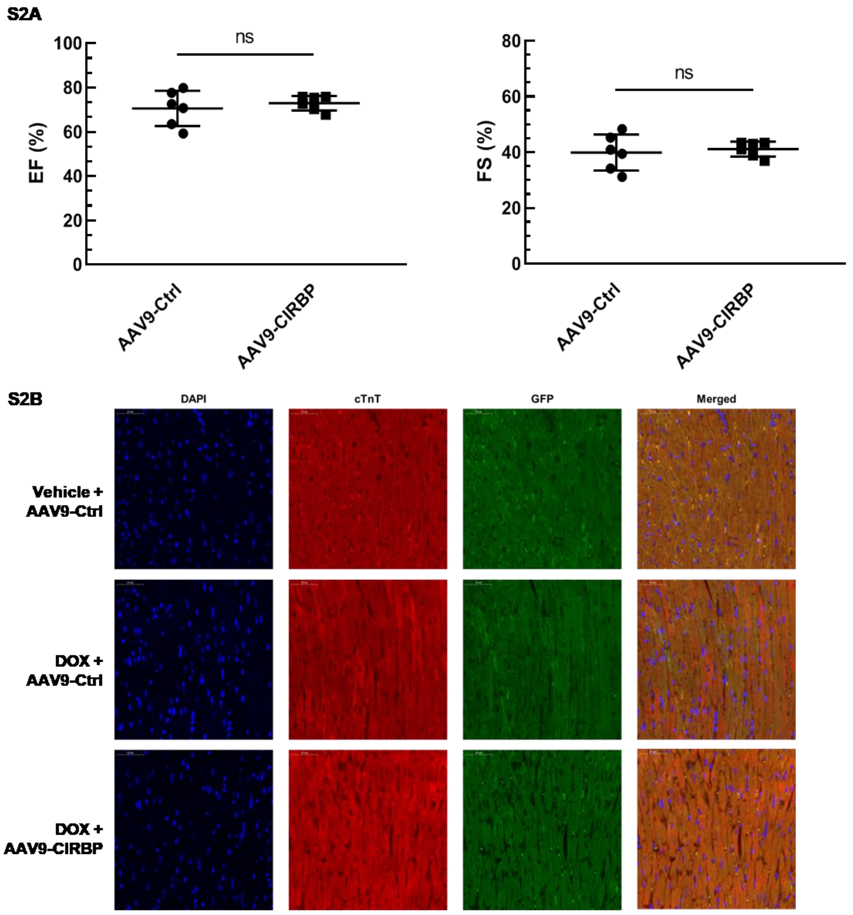

SFigure 2 Cardiomyocyte-specific augmentation of cold-inducible RNA-binding protein (CIRBP) expression has no impact on mouse cardiac function at the baseline level

S2A, Echocardiographic evaluation of cardiac function evaluation in mice preinjected with an adeno-associated virus 9 (AAV9) vector harbouring a control (AAV9-Ctrl)- or CIRBP (AAV9-CIRBP)-expressing cassette. Left, EF; right, FS. n = 6 per group. ns, no significance. n = 6 per group. **S2B**, Representative immunofluorescence images of heart tissue sections from mice injected with an AAV9-Ctrl- or AAV9-CIRBP-expressing cassette in the presence or absence of DOX (vehicle or DOX) verifying the infection efficiency in each group. Nuclei, DAPI (blue); cardiomyocytes, cTnT (red); GFP (green). Scale bar, 50 µm. Statistical significance was analysed using Student's t-test. ns, no significance

Supplementary Figure 3

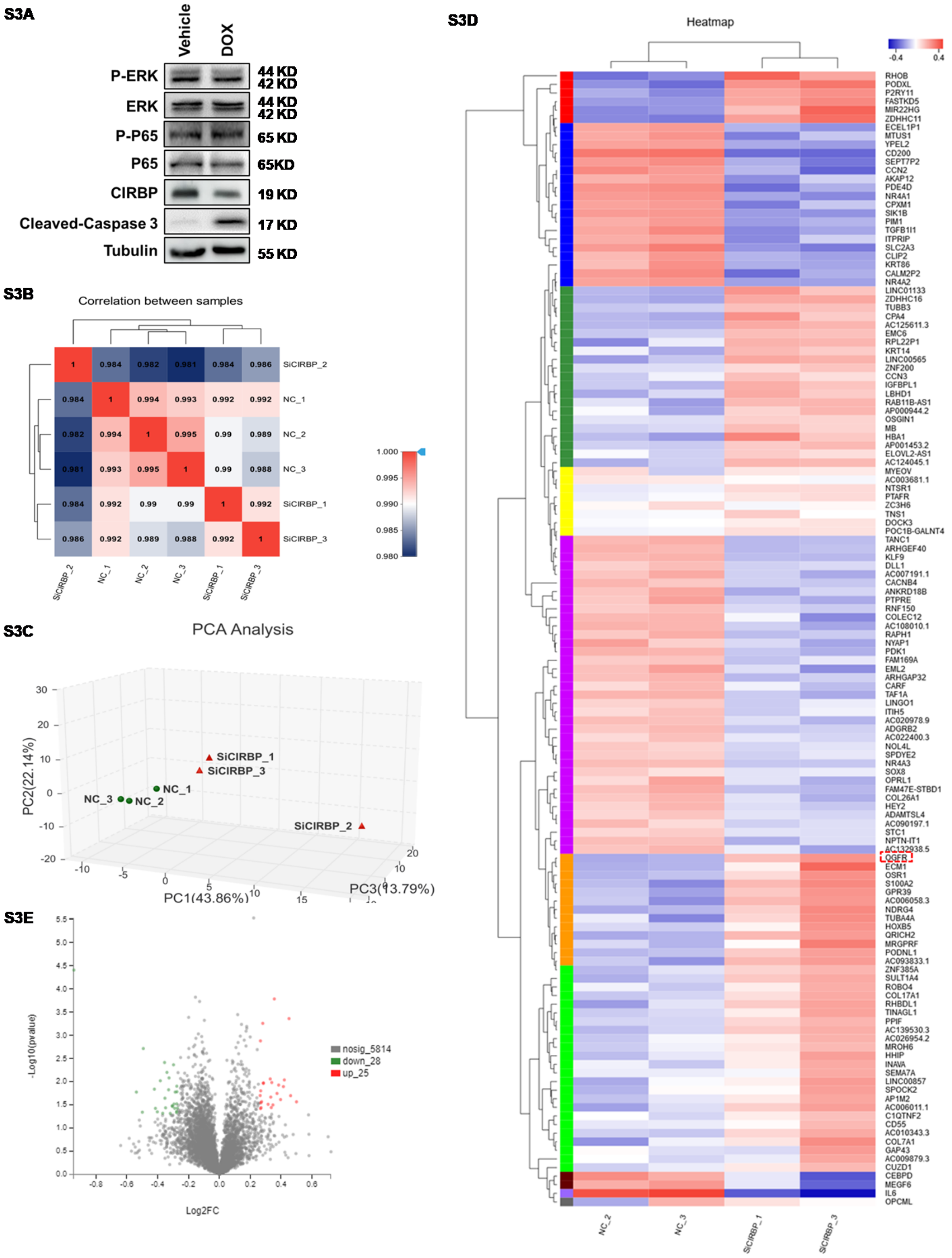

SFigure 3 Identifying CIRBP downstream effectors accounting for cardiomyocyte apoptosis through transcriptomics and proteomics analysis

S3A, Immunoblot analysis of ERK and NF-κB expression in AC16 cells treated with vehicle or DOX. **S3B**, Correlation analysis of RNA-seq data from AC16 cells transfected with negative control (NC) siRNA or CIRBP siRNA (SiCIRBP). **S3C**, Principal component analysis (PCA) of the RNA-seq data described in S3B. **S3D**, Heatmap showing differentially regulated transcripts between the NC siRNA- and SiCIRBP-transfected group identified by transcriptomics analysis. Opioid growth factor receptor (OGFR) is indicated by the red dashed rectangle. **S3E**, Volcano plot showing differentially

expressed proteins between the NC siRNA- and SiCIRBP-transfected groups identified by proteomics analysis.

Supplementary Figure 4

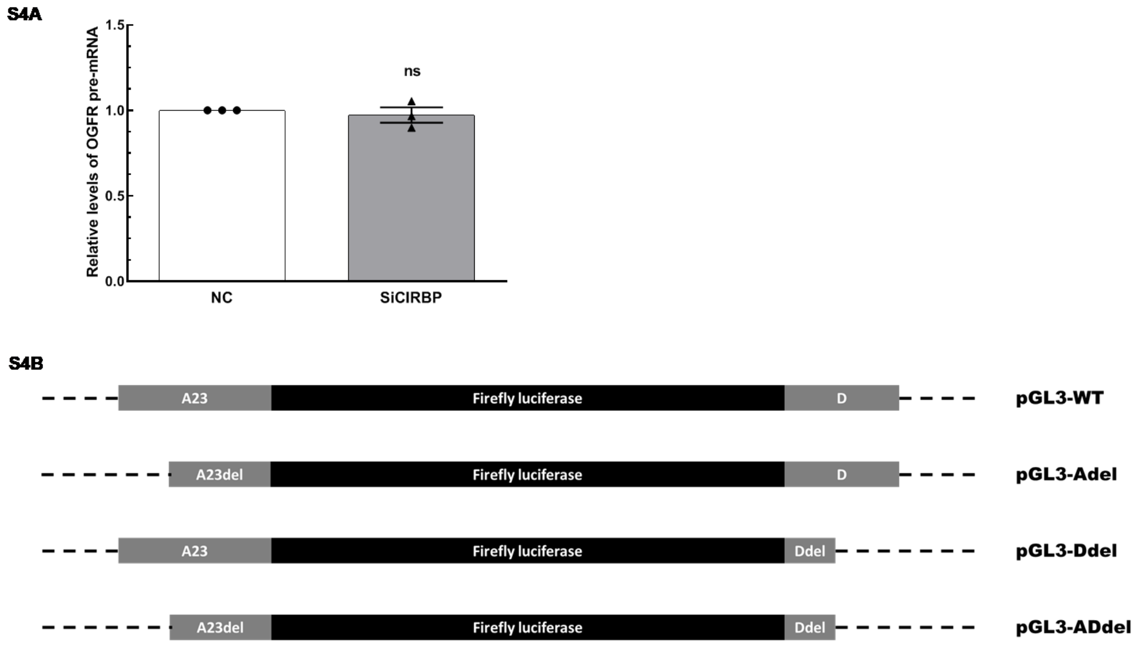

SFigure 4 CIRBP represses OGFR expression by destabilizing OGFR mRNA

S4A, Relative levels of precursor OGFR mRNA in AC16 cells transfected with NC siRNA or SiCIRBP. the data are presented the mean ± SD of 3 independent experiments. Statistical significance was analysed using Student’s t-test. ns, no significance. **S4B**, Schematics of the chimaeric luciferase constructs utilized in Figure 5F.

Supplementary Figure 5

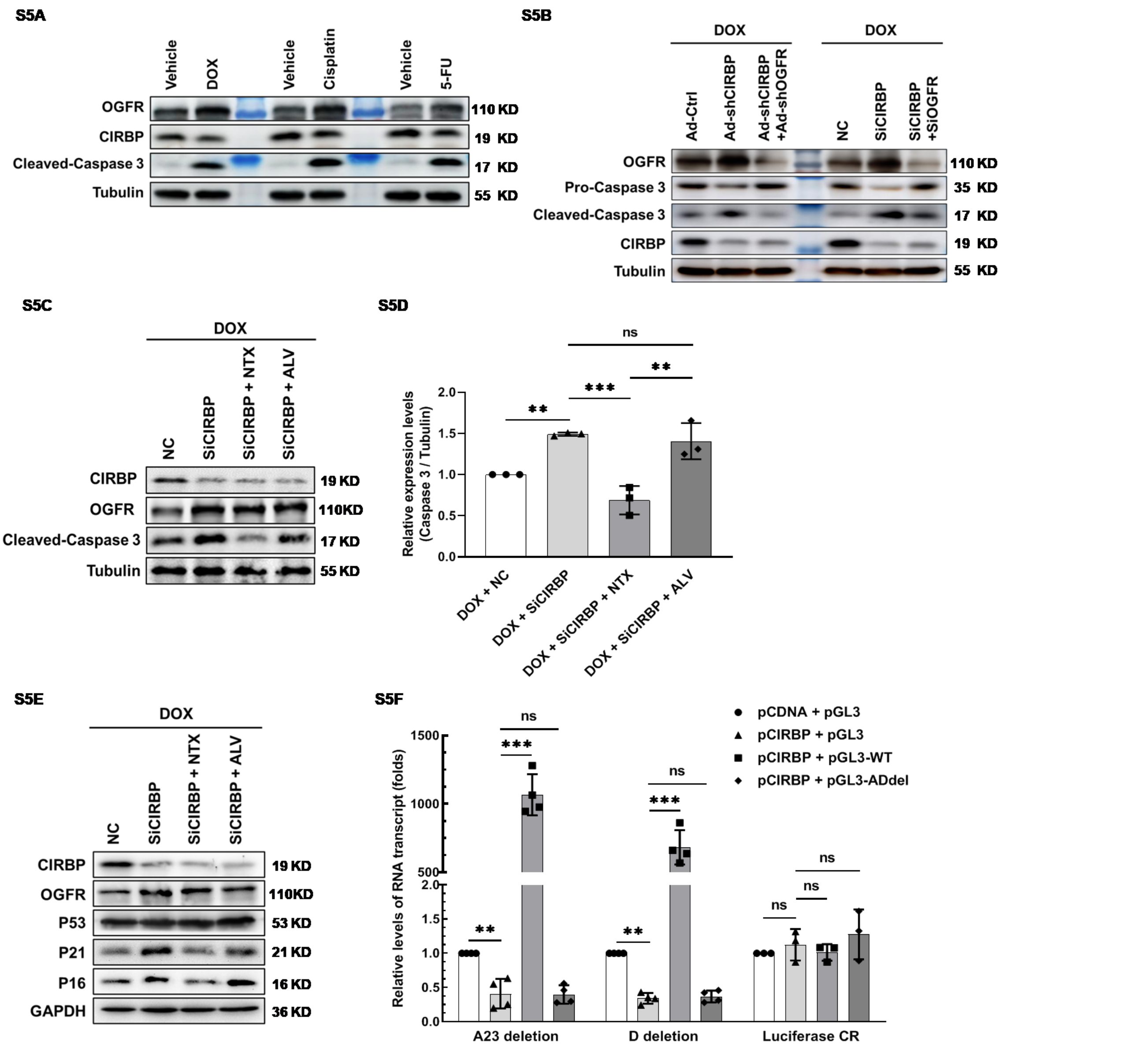

SFigure 5 CIRBP-mediated OGFR repression plays an essential role in protecting cardiomyocytes from apoptosis induced by DOX

S5A, Immunoblot analysis of the expression patterns of CIRBP and OGFR in AC16 cells undergoing apoptosis induced by three different chemotherapeutics (DOX, cisplatin, and 5-FU). **S5B**, Immunoblot analysis of DOX-induced apoptosis in human induced pluripotent stem cell-derived cardiomyocytes (hiPSC-CMs) infected with an adenovirus vector expressing control shRNA (Ad-Ctrl), CIRBP shRNA (Ad-shCIRBP), and shRNAs targeting both CIRBP and OGFR (Ad-shCIRBP + Ad-shOGFR) and in T0519 cells transfected with NC siRNA, SiCIRBP, and siRNAs targeting both CIRBP and OGFR (SiCIRBP + SiOGFR). **S5C**, Representative immunoblots assessing DOX-induced apoptosis in AC16 cells treated with NC siRNA (DOX + NC), SiCIRBP (DOX + SiCIRBP), SiCIRBP plus naltrexone (NTX) (DOX + SiCIRBP + NTX), or SiCIRBP plus alvimopan (ALV) (DOX + SiCIRBP + ALV). **S5D**, Densitometric quantification of relative protein levels of Cleaved-Caspase 3 in S5C. **S5E**, Immunoblot analysis of the activation of opioid growth factor (OGF)/OGFR signalling (p21 and p16 expression changes) in the AC16 cells described in S5C. **S5F**, Real-time qPCR

analysis of the relative transcript levels of related genes in Figure 6G. A23 deletion (n=4), the deleted region in A23 depicted in Figure 5C; D deletion (n=4), the deleted region in D depicted in Figure 5C; luciferase CR (n=3), the coding region of firefly luciferase in all pGL3-derived constructs. The data are presented as the mean ± SD of 3 independent experiments unless otherwise specified. Statistical significance was analysed by one-way ANOVA followed by Tukey–Kramer multiple comparisons. **, $p < 0.01$; ***, $p < 0.001$; ns, no significance.

Supplementary Figure 6

S6A

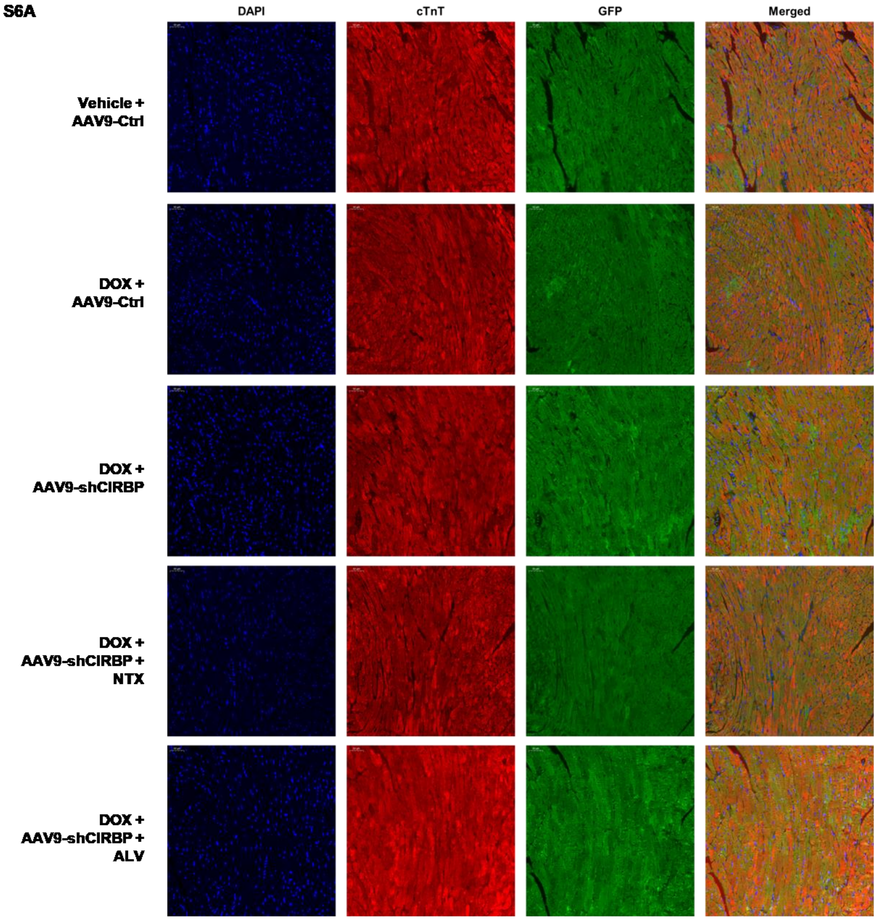

S6B

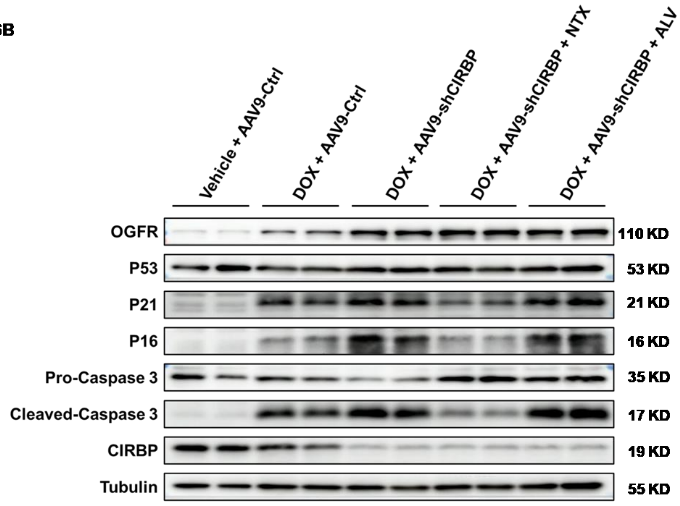

S6C

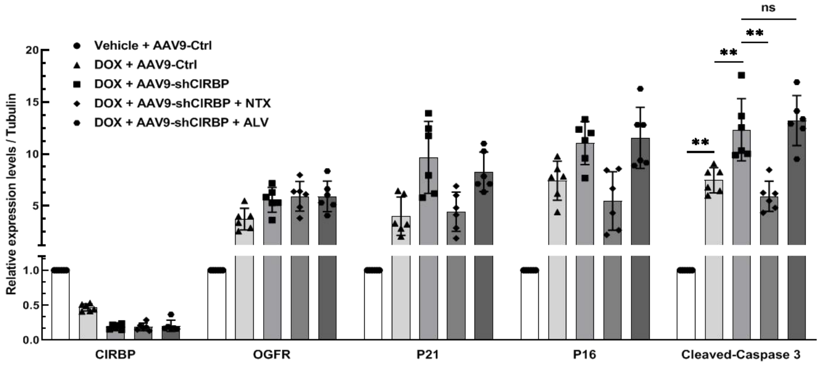

SFigure 6 OGFR blockade ameliorates CIRBP deficiency aggravated myocardial apoptosis and cardiotoxicity during DOX administration

S6A, Representative immunofluorescence images of heart tissue sections from mice injected with an AAV9-Ctrl- or shCIRBP (AAV9-shCIRBP)-expressing cassette in the groups described in Figure 6A verifying the infection efficiency in each group. Nuclei, DAPI (blue); cardiomyocytes, cTnT (red); GFP (green). Scale bar, 50 μm. **S6B**, Representative immunoblots of protein expression in heart tissues from the mice in Figure 6A. **S6C**, Densitometric quantification of CIRBP, OGFR, P21, P16, and Cleaved-Caspase 3 expression levels in S6B. The data are presented as the mean ± SD, n = 6 per group. Statistical significance was analysed by one-way ANOVA followed by Tukey–Kramer multiple comparisons. **, $p < 0.01$; ns, no significant.

Supplementary table legends

Table S1. Differentially expressed transcripts identified by RNA sequencing (RNA-seq) analysis of the negative control (NC) siRNA- and CIRBP siRNA (SiCIRBP)-transfected groups. Upregulated, 171 transcripts, ≥ 2-fold change; downregulated, 216 transcripts, ≤ 0.5-fold change; adjusted p value ≤ 0.05.

Table S2. Differentially expressed proteins identified by iTRAQ proteomics analysis of the NC siRNA- and SiCIRBP-transfected groups. Upregulated, 25 proteins, ≥ 1.2-fold change; downregulated, 28 proteins, ≤ 0.83-fold change; adjusted p value ≤ 0.05.

References

1. Liu Y, Xing J, Li Y, Luo Q, Su Z, Zhang X, et al. Chronic hypoxia-induced Cirbp hypermethylation attenuates hypothermic cardioprotection via down-regulation of ubiquinone biosynthesis. Sci Transl Med. 2019;11(489).
2. Wang X, Tian J, Jiao X, Geng J, Wang R, Liu N, et al. The novel mechanism of anticancer effect on gastric cancer through inducing G0/G1 cell cycle arrest and caspase-dependent apoptosis in vitro and in vivo by methionine enkephalin. Cancer Manag Res. 2018;10:4773-87.
3. Asnani A, Moslehi JJ, Adhikari BB, Baik AH, Beyer AM, de Boer RA, et al. Preclinical Models of Cancer Therapy-Associated Cardiovascular Toxicity: A Scientific Statement From the American Heart Association. Circ Res. 2021;129(1):e21-e34.
4. Li DL, Wang ZV, Ding G, Tan W, Luo X, Criollo A, et al. Doxorubicin Blocks Cardiomyocyte Autophagic Flux by Inhibiting Lysosome Acidification. Circulation. 2016;133(17):1668-87.
5. Yoon JH, Gorospe M. Cross-Linking Immunoprecipitation and qPCR (CLIP-qPCR) Analysis to Map Interactions Between Long Noncoding RNAs and RNA-Binding Proteins. Methods Mol Biol. 2016;1402:11-7.
